# A majority of HIV persistence during antiretroviral therapy is due to infected cell proliferation

**DOI:** 10.1038/s41467-018-06843-5

**Published:** 2018-11-16

**Authors:** Daniel B. Reeves, Elizabeth R. Duke, Thor A. Wagner, Sarah E. Palmer, Adam M. Spivak, Joshua T. Schiffer

**Affiliations:** 1 Vaccine and Infectious Diseases Division, Fred Hutchinson Cancer Research Center, 1100 Fairview Ave., Seattle, WA 98122 USA; 2 Department of Medicine, University of Washington, 1959 NE Pacific St., Seattle, WA 98195 USA; 3 Department of Pediatrics, University of Washington, 1959 NE Pacific St., Seattle, WA 98195 USA; 4 Center for Global Infectious Disease Research, Seattle Children’s Research Institute, 1900 9th Ave., Seattle, WA 98101 USA; 5 Centre for Virus Research, The Westmead Institute for Medical Research, University of Sydney, 176 Hawkesbury Rd., Sydney, NSW 2145 Australia; 6 Department of Medicine, University of Utah, 30N 1900 E, Salt Lake City, UT 84132 USA; 7 Clinical Research Division, Fred Hutchinson Cancer Research Center, 1100 Fairview Ave., Seattle, WA 98122 USA

## Abstract

Antiretroviral therapy (ART) suppresses viral replication in people living with HIV. Yet, infected cells persist for decades on ART and viremia returns if ART is stopped. Persistence has been attributed to viral replication in an ART sanctuary and long-lived and/or proliferating latently infected cells. Using ecological methods and existing data, we infer that >99% of infected cells are members of clonal populations after one year of ART. We reconcile our results with observations from the first months of ART, demonstrating mathematically how a fossil record of historic HIV replication permits observed viral evolution even while most new infected cells arise from proliferation. Together, our results imply cellular proliferation generates a majority of infected cells during ART. Therefore, reducing proliferation could decrease the size of the HIV reservoir and help achieve a functional cure.

## Abstract (web summary)

HIV infected cells persist for decades in patients under ART, but the mechanisms responsible remain unclear. Here, Reeves et al. use modeling approaches adapted from ecology to show that cellular proliferation, rather than viral replication, generates a majority of infected cells during ART.

## Introduction

Antiretroviral therapy (ART) limits HIV replication leading to elimination of most infected CD4+ T cells^1^. Yet, some infected cells persist and are cleared from the body extremely slowly despite decades of treatment^2,3^. There is debate whether infection remains due to HIV replication within a small population of cells^4,5^ or persistence of memory CD4+ T cells with HIV integrated into human chromosomal DNA^3,6,7^. If the latter mechanism predominates, prolonged cellular lifespan and/or cellular proliferation may sustain stable numbers of infected cells.

To optimize HIV cure strategies, mechanisms sustaining infection must be understood. Persistent viral replication in a sanctuary where ART levels are inadequate implies a need to improve ART delivery^8^. If HIV persists without replication as a latent reservoir of memory CD4+ T cells, then survival mechanisms of these cells are ideal therapeutic targets. Infected cell longevity might be addressed by reactivating the HIV replication cycle^9^ and strengthening the anti-HIV immune response, leading to premature cellular demise. Anti-proliferative therapies could limit homeostatic or antigen-driven proliferation^10–12^.

These competing hypotheses have been studied by analyzing HIV evolutionary dynamics. Due to the high mutation rate of HIV reverse transcriptase and large viral population size^13^, HIV replication produces high viral diversity^13–15^. New strains predominate due to continuous positive immunologic selection pressure. Repeated selective sweeps cause genetic divergence, or a positive molecular evolution rate^16^, measured by increasing genetic distance between the consensus and founder virus^17–19^.

One study documented new HIV mutants during months 0–6 of ART in three participants at a rate equivalent to pre-ART. New mutations were noted across multiple anatomic compartments, implying widespread circulation of evolving strains^4^. One proposed explanation was a drug sanctuary in which ART levels were insufficient to stop new infection events. Alternative interpretations were experimental error related to PCR resampling, or variable cellular age structure within the phylogenetic trees^20,21^.

In other studies of participants on ART for at least one year, viral evolution was not observed despite sampling multiple anatomic compartments^22–25^. Identical HIV DNA sequences were noted in samples obtained years apart^14,26,27^, suggesting long-lived latently infected cells as a possible mechanism of persistence^3,6,7,24,25^. Clonal expansions of identical HIV DNA sequences were observed, demonstrating that cellular proliferation generates new infected cells^4,12,24,28–30^. Multiple, equivalent sequences were noted in blood, gut-associated lymphoid tissue (GALT), and lymph nodes, even during the first month of ART^24,29,30^.

The majority of these studies relied on sequencing single HIV genes which may overestimate clonality because mutations in other genome segments could go unobserved^17,31^. These studies also measured total HIV DNA. However, a majority of HIV DNA sequences have deleterious mutations and do not constitute the replication-competent reservoir^32,33^. A recent study utilized whole-genome sequencing to confirm abundant replication-competent sequence clones^34^. In another cohort, rebounding HIV arose from replication-competent clonal populations^35^.

Another approach to define HIV clonality involves sequencing the HIV integration site within human chromosomal DNA^36–40^. While HIV tends to integrate into the same genes^39,41^, it is extremely unlikely that two infection events would result in integration within precisely the same chromosomal locus^37^. Thus, integration site analyses eliminate overestimation of clonality. Previous studies found significant numbers of repeated integration sites, providing strong evidence that these infected cells arose from cellular proliferation^42,43^, though replication competency of the virus was not confirmed^39^. These studies documented equivalent sequences in a minority (<50%) of observed sequences, leading to the conclusion that proliferation only partially drives HIV persistence.

Here, we identify that incomplete sampling leads to underestimation of the true proportion of clonal sequences. Using ecologic tools, we show that nearly all observed unique sequences are actually members of clonal populations derived from cellular proliferation. We predict that the HIV reservoir consists of a small number of massive clones, and a massive number of small clones. We next design a mechanistic mathematical model to reconcile apparent evolution during the early months of ART with clonality after a year of ART. The model includes all proposed mechanisms for HIV persistence including a drug sanctuary, long-lived infected cells, and proliferating infected cells. The model highlights that observed HIV evolution during the first 6 months of ART is caused by sampling long-lived cells that were generated by viral replication. Sampling early during ART detects a positive molecular evolution rate due to the fossil record of past infections rather than current viral replication. After one week of ART, a majority of new infected cells are generated by proliferation. While it is impossible to rule out an unobserved drug sanctuary, our results suggest that cellular proliferation predominantly drives HIV persistence on ART. Consequently, anti-proliferative therapies embody a meaningful approach for HIV cure.

## Results

### Genetic signatures of HIV persistence

**Fig. 1:**
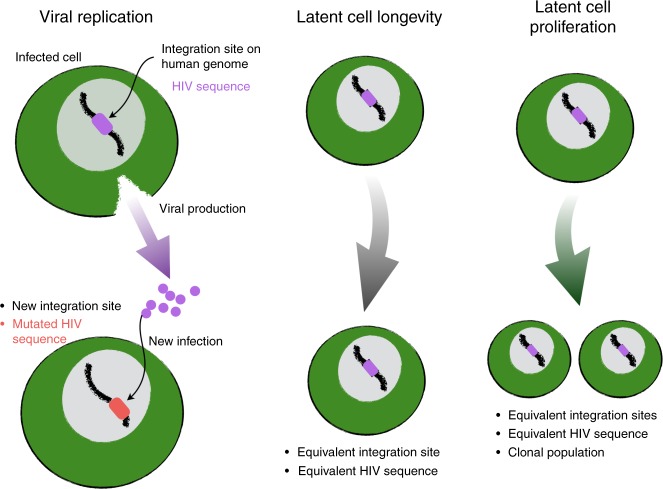
Genetic signatures of HIV persistence during ART. Viral replication despite antiretroviral therapy (ART) would lead to accrual of new mutations (HIV sequence color change) and novel chromosomal integration sites in newly infected cells. Longevity of latently infected cells maintains sequences and integration sites. Cellular proliferation of latently infected cells produces clonal populations carrying identical HIV sequences in identical integration sites

When HIV infects a cell, it integrates its DNA into human chromosomal DNA^44^. A majority of new infected cells are marked by novel integrated HIV sequences and unique integration sites in terms of chromosomal location (Fig. 1) due to the high error rate of HIV reverse transcriptase and the integration process. Thus, a signature suggesting that ongoing viral replication (perhaps due to inadequate drug delivery to certain micro-anatomic regions) sustains the HIV reservoir on ART would be the observation of continual accrual of new mutations during ART.

In a subset of infected CD4+ T cells, HIV replication does not progress beyond chromosomal integration and the virus enters latency^44^. If the same HIV sequences (or integration sites) are found over long-time intervals, this provides a signature that latent cell longevity or proliferation allows HIV to persist. If equivalent HIV sequences with identical chromosomal integration sites are identified in multiple cells, this provides a signature that these sequences were generated via cellular proliferation, rather than HIV replication (Fig. 1).

As another approach, we contrast the impact of HIV replication and cellular proliferation on HIV persistence during ART by quantifying the numbers or fractions of unique and equivalent sequences. Human DNA polymerase has much higher copying fidelity than HIV reverse transcriptase. Thus, we assume cells arising from viral replication will contain unique sequences while cells arising from cellular proliferation will contain equivalent sequences and be members of clonal populations.

### HIV DNA as a marker of replication-competent HIV clonality

Most integrated HIV DNA carries mutations that render the virus replication incompetent. Quantification of total HIV DNA overestimates the size of the replication-competent reservoir by 2–3 orders of magnitude relative to viral outgrowth assays^32^. Replication incompetent, equivalent HIV sequences are commonly present in multiple cells^24,29^. Because these sequences are terminally mutated, they are concrete evidence that another mechanism (cellular proliferation) copies HIV DNA. The proportion of clonal sequences is similar when analysis includes only replication-competent sequences, or all HIV DNA^34^. Therefore, while total HIV DNA may not predict quantity of replication-competent viruses, estimates of clonal frequency using total HIV DNA may be extrapolated to the replication-competent reservoir^33^. We use total HIV DNA as it allows a greater sample size for analysis.

### Clonal HIV DNA and replication-competent HIV during ART

To examine the clonal structure of total and replication-competent HIV DNA, we ranked observed sequences from several studies according to their abundance. So-called rank-abundance curves are ordered histograms denoted *a*(*r*) such that *a*(1) is the abundance of the largest clone. These curves facilitate identification of the richness *R* = max(*r*), sample size $N = \mathop {\sum }\nolimits_r a\left( r \right)$, and number of singletons $N_1 = \mathop {\sum }\nolimits_r I[a(r) = 1]$. Here *I*[·] is the indicator function equal to 1 when its argument is true and 0 otherwise.

**Fig. 2:**
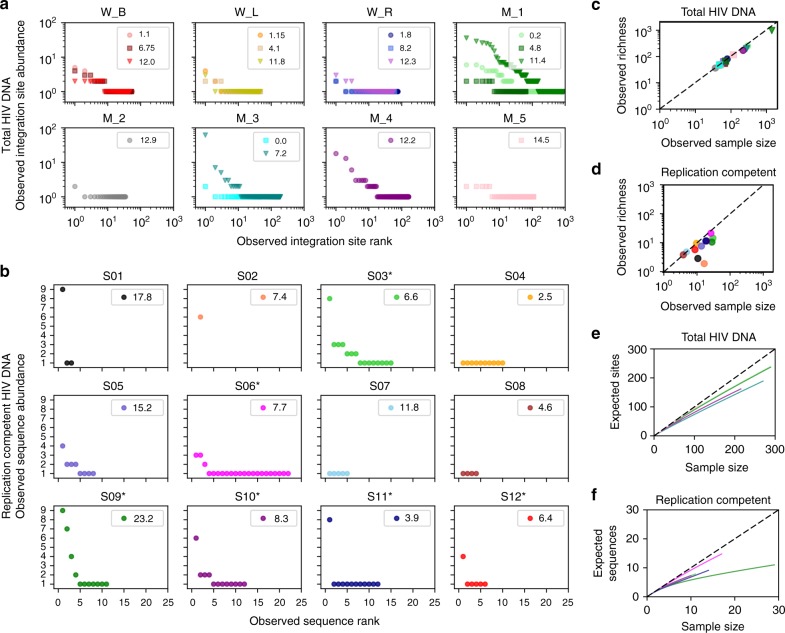
Evidence for clonal HIV sequences. **a** Total HIV DNA from integration site data^36,37^ arranged as rank-abundance curves. Each panel represents a participant, and each curve the time point during ART (indicated in years in the panel legend). W and M in the panel headings distinguish the study. **b** Similar rank-abundance curves for replication-competent HIV DNA^34^. Each panel represents a participant. Data used for analysis in Figs. 3 and 5 (noted by asterisks in panel titles) have sample size *N* > 20 sequences. **c**, **d** Sample sizes of total HIV DNA (**c**) and replication-competent HIV DNA (**d**) at each participant time point plotted against corresponding observed sequence richness. For all HIV DNA data and replication-competent HIV DNA data with sufficient sampling (*N* > 20), the observed richness is less than the sample size (below the dotted line *y* = *x*), owing to the presence of sequence clones. Observed richness correlates with sample size, indicating further sampling consistently uncovers new sequences. **e**, **f** Sample rarefaction curves for all 17 time points from the 8 study participants from **a** and five sufficiently sampled study participants from **b**. Rarefaction demonstrates the number of distinct integration sites or HIV sequences expected from a given sample size. For both data sources, at low sample size, distinct sequences are expected from each new sample. As sample size increases, distinct sequences are increasingly less likely to be detected owing to the presence of repeatedly detected sequence clones. Thus, curves increasingly flatten until all unique sequences are detected and the curve is completely flat. All colors correspond to data in **a** and **b**

Wagner et al.^37^ sampled HIV DNA in three participants at three time points 1.1–12.3 years following ART initiation. Maldarelli et al.^36^ sampled HIV DNA from five participants at one to three time points 0.2–14.5 years following ART initiation. In these studies, 1–16% (mean: 7%) of sequences were members of observed sequence clones (Fig. 2a)^36,37^, meaning that HIV DNA was in the same chromosomal integration site in at least two cells. The absolute number of observed sequence clones *N*_*i*>1_ in the 17 samples ranged from 1–150 (mean: 15). The remaining sequences were identified in a specific chromosomal integration site in only one cell (observed singletons)^37^. For total HIV DNA, at each participant time point, certain sequences predominated: the largest observed clone contained 2–62 sequences (mean: 11), accounting for 3–26% (mean: 9%) of total observed sequences.

Hosmane et al.^34^ sequenced replication-competent HIV isolates from 12 study participants on ART: 0–28% (mean: 11%) of sequences were members of observed sequence clones (Fig. 2b). Three participants did lack clones, which might reflect low sequence sample size. As a result, we excluded participants with fewer than 20 total sequences from individual analyses but did include these data for population level evaluations. For replication-competent HIV in the five individuals having sequence sample size *N* > 20, certain sequences dominated: the largest observed sequence clone contained 3–9 sequences (mean: 6.8), accounting for 11–42% (mean = 28%) of total observed sequences. The number of non-singleton sequence clones *N*_*i*>1_ in the five samples ranged from 1–7 (mean: 3.8).

### Low sampling depth relative to total sequence population

Larger sample sizes exist for total HIV DNA (Fig. 2c) than for replication-competent HIV (Fig. 2d). Both for total HIV DNA and the five included replication-competent data sets (where *N* > 20), the number of observed unique sequences (*R*^obs^ or observed sequence richness) was always less than *N* (Fig. 2c, d). In both cases, observed richness (*R*^obs^) correlated with sequence sample size (*N*) (Fig. 2c, d). Thus, we infer that further sampling would uncover new unique sequences. To quantify the relationship between sample size and discovery, we calculated rarefaction curves (Fig. 2e, f: see Methods and Supplementary Methods for details). These curves relate expected sequence discovery and sample size. At low sample size, each additional sample likely uncovers a new sequence. As sampling increases, the chance of sampling a previously documented sequence increases, and the slope of the rarefaction curve begins to flatten. As sample size approaches the true population richness, the curve plateaus and few new unique sequences remain unsampled. Current sampling depth remains on the steep, initial portion of the curve (Fig. 2c, d).

### Lower bounds of true HIV sequence richness

**Fig. 3:**
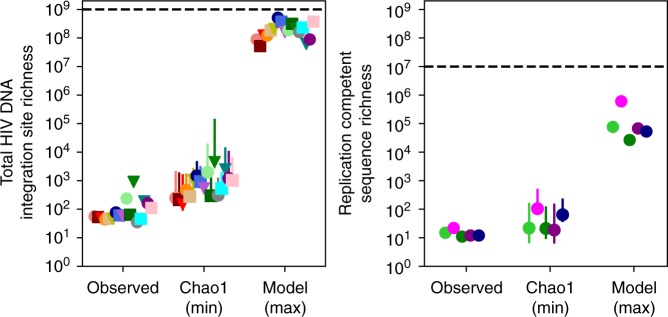
Observations underestimate the number of distinct HIV sequences during ART. Observed sequence richness underestimates the true HIV sequence richness. For both data sources, Chao1 provides an estimate of the lower bound (min) of true sequence richness (error bars are asymmetric confidence intervals, see Supplementary Methods). In all cases, Chao1 estimates are above observed values. Our conservative modeling technique estimates a much higher upper bound (max) for true sequence richness. Nevertheless, the total HIV sequence population size (dashed lines: 10^9^ for total HIV DNA and 10^7^ for replication-competent HIV) is 1–2 orders of magnitude above the upper bound estimates for sequence richness, suggesting substantial clonality of HIV sequences. All marker colors correspond to data in Fig. 2a and b

To estimate a lower bound for true sequence richness, we used the Chao1 estimator, a nonparametric ecologic tool that uses frequency ratios of observed singletons *N*_1_ and doubletons *N*_2_ (see Methods and Supplementary Methods)^45,46^. For the HIV reservoir, theoretical values for true richness range from one (if all sequences are identical and originate from a single proliferative cell) to the total population size (if all sequences are distinct and originate from error-prone viral replication). Estimated lower bounds for true sequence richness exceeded observed richness, typically by an order of magnitude in both total HIV DNA and replication-competent HIV (Fig. 3). These lower bound estimates for sequence richness are far lower than previously estimated population sizes for HIV DNA and replication-competent HIV DNA sequences^2,3,6^, suggesting that clones may predominate. Asymmetric confidence intervals for these estimates are detailed in the Supplementary Methods.

### A majority of observed sequences are clonal

The Chao1 estimator does not integrate information about the total population size. However, estimates for the total number of total HIV DNA and replication-competent sequences in the entire reservoir exist^33^. Using that additional information, we developed an ecologic model to extrapolate the true rank-abundance of HIV sequences for each participant time point.

Based on the observation that observed data was roughly log-log-linear (Fig. 2a), we chose a power-law model for rank-abundance: *a*(*r*) ∝ *r*^−*α*^. Other simple functional forms were explored (exponential, linear, and biphasic power-law) but were worse or equivalent for data fitting. Our model requires three parameters: the power-law exponent (*α*), the sequence population size (*L*), and the sequence richness (*R*). Model fitting is described in the Methods with additional detail in the Supplementary Methods. Briefly, we generated 2500 possible models for each data set, choosing a plausible fixed population size from available data (*L* = 10^9^ for HIV DNA and *L* = 10^7^ for intact, replication-competent HIV DNA)^2,3,6,33,47^. We then recapitulated the experiment by taking *N* random samples from each model distribution and comparing sampled data to experimental data to find optimal model parameters. This resampling method correctly inferred the power-law exponent from simulated power-law data (Supplementary Fig. 1).

For experimental data, we could not precisely identify *R*. Recognizing this uncertainty, we developed an integral approximation to estimate the largest possible richness (least clonality) given *L* and the best-fit *α* (derivation in Supplementary Methods and illustration in Supplementary Fig. 2). Using the lower bound estimate from the Chao1 estimator, we were able to fully constrain the estimate of true HIV sequence richness in the reservoir. Our maximal estimates for sequence richness were notably several orders of magnitudes higher than Chao1 estimates (Fig. 3) but lower than the total sequence population size (*L*).

**Fig. 4:**
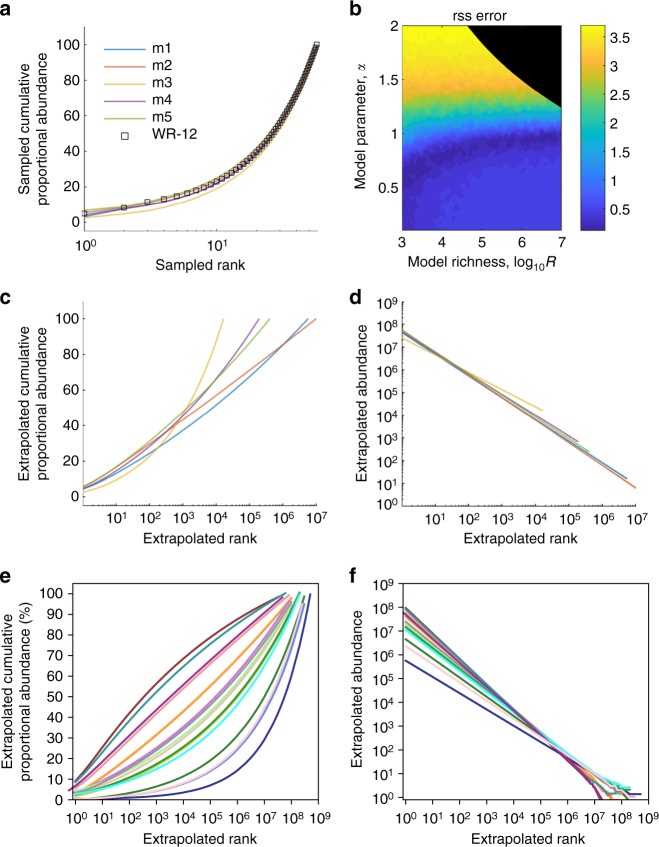
Ecologic modeling suggests a majority of HIV DNA sequences are clonal. To describe the true rank-abundance distribution of the HIV reservoir, we used a power-law model and recapitulated experimental sampling (sample size equal to the experimental sample size) from 2500 theoretical power-law distributions to fit the best model to participant data in Fig. 2a. Theoretical distributions varied according to the slope of the power-law and the true sequence richness but were fixed at 10^9^ total HIV DNA sequences. **a** Five best model fits (m1–5) to cumulative proportional abundance curves from a single representative participant time point (black circles: WR, 12 years on ART). **b** Heat map representing model fit (dark blue optimal) according to power-law exponent *α* and true sequence richness *R*. Black shaded area represents parameter sets excluded based on mathematical constraints of the power-law (upper bound on sequence richness). A wide range of values for sequence richness allow excellent model fit while the power-law exponent exponent is well-defined. **c**, **d** Extrapolations of five best models for the participant time point to a reservoir size of 10^9^ cells carrying integrated HIV DNA. **c** Cumulative proportional abundances show that 10^4^ to 10^7^ clones constitute the entire reservoir. **d** Rank-abundance curves show the largest 1000 clones consist of >10^4^ cells each. **e**, **f** Extrapolations of the maximum richness best-fit model for each participant time point (colored to match Fig. 2a) to a total HIV DNA reservoir size of 10^9^ cells. **e** For each participant time point, even with the maximum possible sequence richness, we note a predominance of sequence clones. 50% of the reservoir may be held in the top 200 to 20 million clones. **f** A small number of massive clones (top 1000 clones) each consist of >10^4^ cells and a massive number of smaller clones (~10^7^) each consist of many fewer cells (<100)

**Fig. 5:**
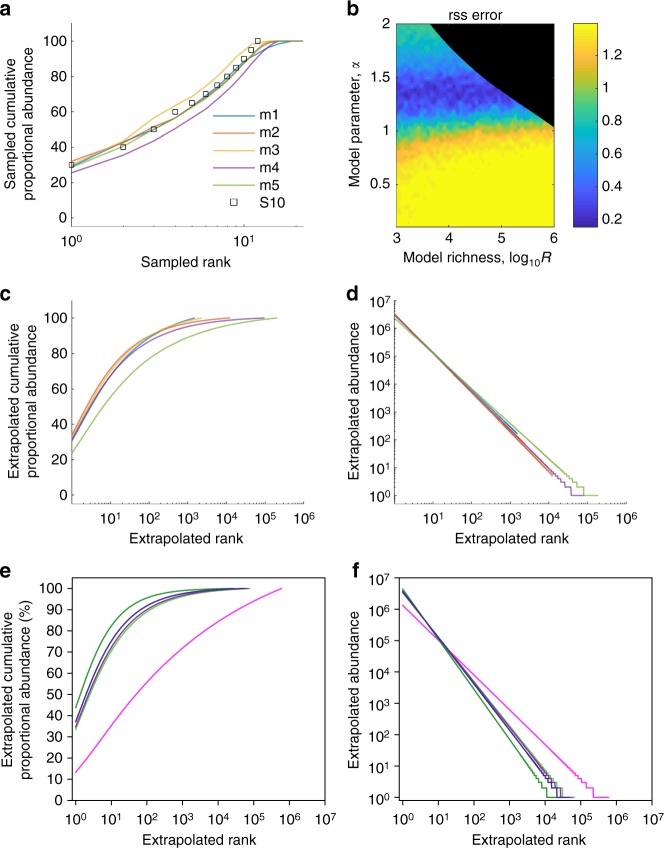
Ecologic modeling suggests a majority of replication-competent HIV sequences are clonal. To describe the true rank-abundance distribution of the HIV reservoir, we used a power-law model and recapitulated experimental sampling (sample size equal to the experimental sample size) from 2500 theoretical power-law distributions to fit the best model to participant data in Fig. 2b. Theoretical distributions varied according to the slope of the power-law and the true sequence richness but were fixed at 10^7^ replication-competent HIV DNA sequences. **a** Five best model fits (m1–5) to cumulative proportional abundance curves from a single representative participant (black circles: S10). **b** Heat map representing model fit (dark blue optimal) according to power-law exponent *α* and true sequence richness *R*. Black shaded area represents parameter sets excluded based on mathematical constraints of the power-law (upper bound on sequence richness). A wide range of values for sequence richness allow excellent model fit while power-law exponent is well-defined. **c**, **d** Extrapolations of five best models for a single participant to a reservoir size of 10^7^ cells carrying replication-competent HIV. **c** Cumulative proportional abundances show that the top 200,000 ranked clones constitute the entire reservoir. **d** Rank-abundance curves show the top 100 clones consist of >2000 cells each. **e**, **f** Extrapolations of the maximum richness best-fit model for each sufficiently sampled participant (colored to match Fig. 2b) to a replication-competent reservoir size of 10^7^ cells. **e** For each participant, even with the maximum possible sequence richness, we note a predominance of sequence clones. 50% of the reservoir may be held in the top 2 to 20 clones. **f** A small number of massive clones (top 100 clones) each consist of >10^3^ cells and a massive number of smaller clones (~10^5^) each consist of many fewer cells (<100)

Our method demonstrated excellent fit to cumulative proportional abundances of observed clones for total HIV DNA (Fig. 4a) and replication-competent HIV DNA (Fig. 5a). For total HIV DNA (Fig. 4b) and replication-competent HIV DNA (Fig. 5b), optimal fit was noted within narrow ranges for the power-law slope parameter but across a wide range of true sequence richness. Using the five best-fit models, we generated extrapolated distributions of the entire HIV sequence rank-abundance for all participant time points. For the participant in Fig. 4c, between 10^4^ and 10^7^ clones were needed to reach 100% cumulative abundance. The ratio of these estimates of true sequence richness to the total number of infected cells with HIV DNA (10^9^), or *R*/*L*, represents an upper bound on the fraction of sequences that are true singletons: we estimate that >99% of infected cells contain true clonal sequences. Similarly, the ratio of estimated true sequence richness to the total number of infected cells with replication-competent HIV for the participant in Fig. 5c was 10^5^/10^7^. Hence, at least 99% of cells are members of clonal populations. Of note, these ratios are relatively stable regardless of assumed reservoir size. For instance, if we assume a true reservoir size of 10^6^, then our estimate of true sequence richness is ~10^4^. In these examples, the best-fit models gave similar estimates for the population size of the largest clones—those accounting for ~50% of the reservoir—10^3^ to 10^4^ clones for HIV DNA in Fig. 4c and 2–20 clones for replication-competent HIV DNA in Fig. 5c. However, the tail of the reservoir, which consists of thousands of smaller clones, could vary considerably across parameter sets with 10^4^ to 10^6^ possible clones accounting for 90% of total HIV DNA and 10^2^ to 10^4^ possible clones accounting for 90% of replication-competent HIV. This variability reflects the fact that true sequence richness is only partially identifiable using our procedure. The extrapolated rank-abundances for each representative data set are presented in Figs. 4d and 5d.

We applied our model fitting procedure to all data in Fig. 2a and sufficiently sampled data in Fig. 2b (see asterisks). We biased against a clonally dominated reservoir to the greatest extent possible by selecting the best fitting power-law exponent and then calculating the maximum possible sequence richness (see details in Supplementary Information and Supplementary Fig. 2). For all cases, even under this conservative assumption, the vast majority of sequences were predicted to be members of true sequence clones. The power-law exponent was lower for HIV DNA (*α* = 0.9 ± 0.1) than for replication-competent HIV DNA (*α* = 1.4 ± 0.2) on average. Here, errors (±) represent standard deviation across 17 and 5 participant data sets, respectively. As a result, the predicted cumulative distribution of HIV DNA (Fig. 4e) was often concave-up with log rank as compared to concave-down with log rank noted for replication-competent HIV DNA (Fig. 5e), suggesting that a smaller number of extremely large clones might make up a higher proportion of the replication-competent HIV reservoir.

For both total HIV DNA (Fig. 4f) and replication-competent sequences (Fig. 5f), the top 100 clones in all participants are estimated to be massive (>10^5^ and >10^4^ associated cells, respectively). However, there are also massive numbers of smaller clones with fewer than 1000 associated cells (>10^7^ and >10^4^, respectively). In contrast to observed data, a majority of sequences are clonal, suggesting that proliferation is the major generative mechanism of persistent HIV-infected cells.

### Modeling combined population data

To increase sample size and eliminate bias related to excluding participants with low sample sizes, we combined results from all participant time points for HIV DNA (17 time points) and replication-competent HIV (12 time points) into single rank order distribution curves. We then fit the power-law models to both sets of data (Supplementary Fig. 3A, B, E, F). We again noted a narrow range of possible values for the power-law exponent and a large range of possible values for true sequence richness. The exponent was again *α* < 1 for total HIV DNA and *α* ≈ 1 for replication-competent virus (Supplementary Fig. 3A, E), leading to concave-up and linear relationships between log cumulative proportional abundance and log rank, respectively (Supplementary Fig. 3C, G). We estimated that at least 99.9% of total HIV DNA (Supplementary Fig. 3C) or replication-competent HIV (Supplementary Fig. 3G) contain true clonal sequences. The top 100 HIV DNA clones (Supplementary Fig. 3D) and replication-competent clones (Supplementary Fig. 3H) contained >10^6^ and >10^4^ associated cells, respectively.

Using the population level data, we generated sample rarefaction curves from the extrapolated rank-abundance curves. These curves show that after 10,000 sequences were sampled, the observed sequence richness would continue to increase with more sampling (Supplementary Fig. 4). Even if experimental sample sizes could be increased 100-fold from the present data, sequences would likely continue to be dominated by those from large clones. Our methods, or other inference techniques, may therefore be necessary to realistically estimate the clonal distribution of the HIV reservoir.

### Mathematical model of persistent infected cell dynamics

**Fig. 6:**
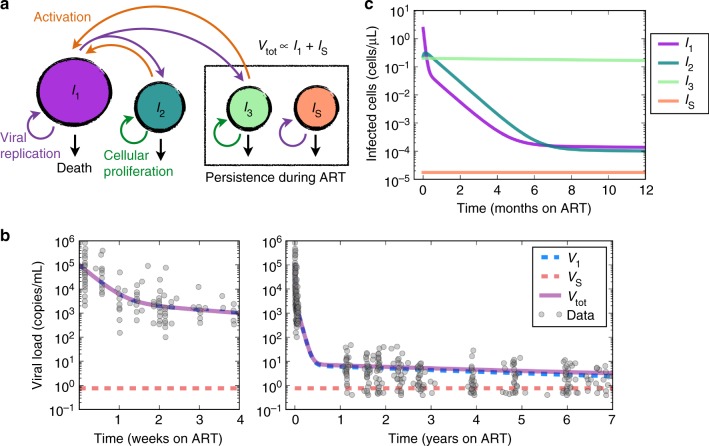
Mechanistic modeling of HIV RNA decay during ART. **a** Model schematic: *I*_1_ cells produce virus, pre-integration latent cells *I*_2_ are longer lived and transition to *I*_1_, and long-lived latently infected cells *I*_3(*j*)_ proliferate and die at measured rates depending on cell phenotype *j* (e.g., effector memory, central memory, naive). Sanctuary cells *I*_S_ allow ongoing HIV replication despite ART. Parameters and their values are discussed in the Methods and listed in Table 1. **b** The mathematical model recapitulates observed HIV RNA data^51^ over weeks and years of ART. *V*_1_ is virus derived from *I*_1_ while *V*_S_ is derived from *I*_S_. **c** *I*_3_ becomes the predominant infected cell state early during ART. *I*_S_ is constrained to be very small to explain the lack of detectable viremia on fully suppressive ART. Lines are colored to match schematic in **a**

Our analyses above identify the critical role of cellular proliferation in generating infected cells after a year of ART but do not capture the dynamic mechanisms underlying this observation or explain possible evidence of viral evolution during months 0–6 of ART^4^. We therefore developed a viral dynamic mathematical model. Our model (Fig. 6a) consists of differential equations, described in detail in the Methods. Most model parameter values are obtained from the literature (Table 1).

**Table 1:** Model parameters

Parameter	Value	Meaning	Units	Source
*R*_0_	8	Basic reproductive number of HIV	[]	^69^
*β*_0_	2×10^−4^	Viral infectivity, used in $\beta _{\it{\epsilon }} = \beta _0(1 - {\it{\epsilon }})$	[μL copy^−1^ day^−1^]	^61, 70, 71^
${\it{\epsilon }}$	0.95	ART efficacy outside the sanctuary	[]	^71, 72^
*π*	10^3^	Viral production rate, used in *n* = *π*/*γ*	[μL copy^−1^ day^−1^]	^61, 70, 71^
*γ*	23	Viral clearance rate, used in *n* = *π*/*γ*	[day^−1^]	^73^
*α*_S_	150	Susceptible cell production rate	[μL copy^−1^ day^−1^]	^54, 70, 71^
*δ*_S_	0.2	Susceptible cell death rate	[day^−1^]	^61, 70, 71^
*δ*_1_	0.8	Productively infected cell (*I*_1_) clearance rate	[day^−1^]	^71, 74^
*δ*_2_	0.02	Pre-integration cell (*I*_2_) death rate	[day^−1^]	^48, 50^
*α*_2_	0.047	Pre-integration cell proliferation rate	[day^−1^]	^42^
*ξ*_2_	0.08	Pre-integration cell activation rate	[day^−1^]	Fit
*α*_3( *j*)_	[0.047,0.015, 0.002]	Proliferation rate of latently infected cells *j* ∈ [T_em_, T_cm_, T_n_] phenotypes, respectively	[day^−1^]	^42^
*ξ*_3_	0.0003	Latent cell activation rate (for all *j*)	[day^−1^]	Fit
*δ*_3( *j*)_		Calculated from latent clearance rate as *θ*_L_ = *α*_3(*j*)_ − *δ*_3(*j*)_ − *ξ*_3_ where *θ*_L_ = − 5.2 × 10^−4^	[day^−1^]	^2, 3^
*τ*_*i*( *j*)_	[1, 10^−2^, 10^−4^*ρ*_*j*_]	Probability of infection of each compartment, taken from *y*-intercepts in ref.^50^	[]	^51^
*ρ*_*j*_	[0.2, 0.75, 0.05]	Fraction of latent infected cells of each phenotype (e.g., from patient #5 in ref.^12^)	[cells μL^−1^]	^12^
*V*(0)	10^2^	Initial viral load (from typical set-point value 10^5^ copies/mL)	[copy μL^−1^]	^69^
*I*_1_(0)	2	Initial concentration of productively infected cells, calculated from *I*_1_(0) = *V*(0)/*n*	[cells μL^−1^]	^75^
*I*_2_(0)	0.2	Initial concentration of pre-integration infected cells	[cells μL^−1^]	^75^
*I*_3(*j*)_(0)	0.2*ρ*_*j*_	Initial concentration of each latent phenotype, calculated from ~10^6^ latently infected cells in ~5 L of blood	[cells μL^−1^]	^2, 12^
*I*_S_(0)	180	Initial concentration of sanctuary cells, calculated from equilibrium model $I_{\mathrm{S}}(0) = \frac{{\alpha _{\mathrm{S}}}}{{\delta _1}} - \frac{{\delta _{\mathrm{S}}}}{{n\beta _0(1 - {\it{\epsilon }}_{\mathrm{S}})}}$, e.g., ref.^56^ SI	[cells μL^−1^]	Calc
*ζ*	0.007	Decay rate of T cell activation	[day^−1^]	^52^
${\it{\epsilon }}_{\mathrm{S}}$	0	ART efficacy in the sanctuary, minimum value	[]	Min
*φ*_S_	10^−5^	Fraction of cells in sanctuary	[]	^4^

Briefly, we classify rapid death *δ*_1_ and viral production within actively infected cells *I*_1_. Cells with longer half-life *I*_2_ are activated to *I*_1_ at rate *ξ*_2_. *I*_2_ may represent CD4+ T cells with a prolonged pre-integration phase, but their precise biology does not affect model outcomes^48^. The state *I*_3(*j*)_represents latently infected cells. We assume each cell carries a single chromosomally integrated HIV DNA provirus^44^. The probabilities of a newly infected cell entering *I*_1_, *I*_2_, *I*_3(*j*)_, are *τ*_1_, *τ*_2_, *τ*_3(*j*)_. Because we are focused on the role of proliferation, we include CD4+ T cell subsets^12^ including effector memory (T_em_), central memory (T_cm_), and naive (T_n_) CD4+ T cells, which have been experimentally proven to turn over at different rates *α*_3(*j*)_, *δ*_3(*j*)_^12,42,43^. We assume all subsets *I*_3(*j*)_ reactivate to *I*_1_ at rate *ξ*_3_^49^.

We allow ART potency ${\it{\epsilon }} \in [0,1]$ to decrease viral infectivity^50^. Other dynamic features of infection such as death rate of infected cells and latent cell proliferation and reactivation rates are unchanged on ART. On ART, the basic reproductive number becomes $R_0\left( {1 - {\it{\epsilon }}} \right) < 1$ when ${\it{\epsilon }} > 0.95$. In a completely susceptible population a reproductive number <1 implies each cell infects fewer than one other cell on average and viral loads decline from steady state. In this setting, only short stochastic cycles of viral replication can occur.

To make a model inclusive of viral evolution despite ART, we allow for the possibility of a drug sanctuary state (*I*_S_) that reproduces with reproductive number $R_0\left( {1 - {\it{\epsilon }}_{\mathrm{S}}} \right) \approx 8$. In the drug sanctuary, ART potency is negligible $({\it{\epsilon }}_{\mathrm{S}} \approx 0)$ such that the sanctuary reproductive number is equivalent to the value from a model without ART. However, target cell limitation or a local immune response causes a sanctuary viral set point to prevent infected cells and viral load from growing unabated. In the absence of contradictory information, we assumed homogeneous mixing of *V*_1_ and *V*_S_ in blood and lymph nodes^4^. Thus, we find that the sanctuary size must be limited to 0.001–0.01% of the original burden of replicating HIV to achieve realistic viral decay kinetics (Fig. 6b)^51^.

Based on the observation that activated, uninfected CD4+ T cells (S), the targets for replicating HIV, decrease in numbers after initiation of ART we also perform a subset of model simulations with a slow target cell decline within the HIV drug sanctuary. We approximate this process with an exponential decay of target cells with rate *ζ* (per day)^52,53^. The decay rate is lower than concurrent decay rates measured from HIV RNA^50,51,54^ because abnormal T cell activation persists for more than a year after ART^53^.

### Accurate simulation of HIV dynamics during ART

**Fig. 7:**
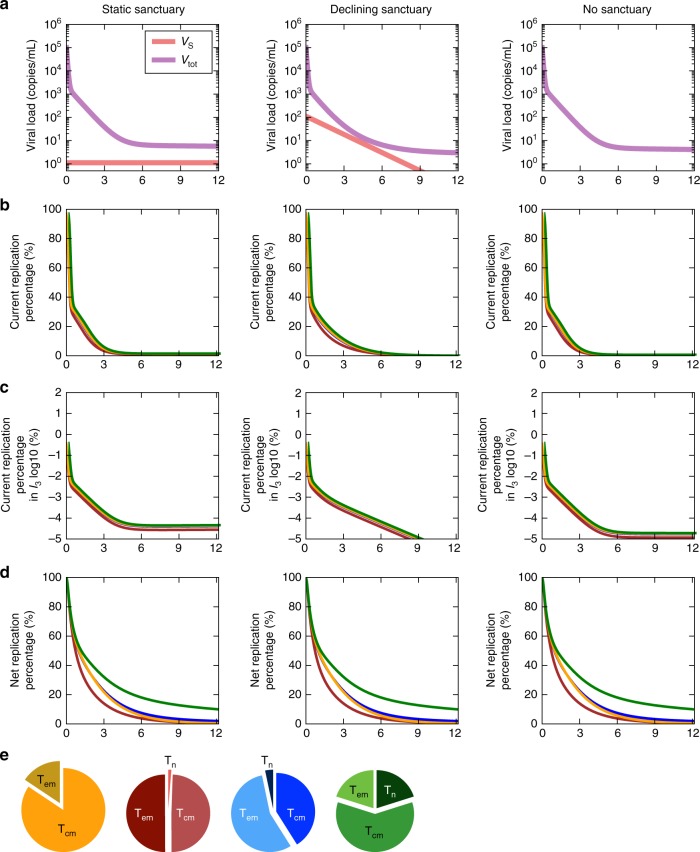
Most infected cells are generated via proliferation within 6 months of ART initiation. Model simulations contrast the number of cells generated by viral replication with those generated by cellular proliferation. The fraction of cells that arose due to viral replication at a time point is referred to as the current replication percentage. The fraction of cells remaining that arose at any time due to viral replication is referred to as the net replication percentage. Simulations are identical except for different assumptions regarding a drug sanctuary (*I*_S_) in each column. **a** Moving left to right, we assume a static drug sanctuary, a slowly declining drug sanctuary and no drug sanctuary. **b** Under all assumptions, once ART is initiated, most current infected cells arise due to cellular proliferation as opposed to HIV replication after 12 months of ART. **c** Current latently infected reservoir cells (*I*_3_) are generated almost entirely by proliferation soon after ART is initiated under all conditions. **d** The fraction of cells that remain that were generated by replication at any time (net) overestimates the fraction generated current percentage during the first 6 months of ART—a trend that is more notable when the reservoir contains a higher proportion of slowly proliferating naive T cells. Importantly, this is the quantity that would be observed experimentally (see Fig. 8). **e** Pie charts indicate reservoir compositions of T cell subsets from published data and correspond with colored lines in **a**–**d**

We fit the model to ultra-sensitive viral load measurements collected from multiple participants in Palmer et al.^51^. We included experimentally derived values for most parameter values (Table 1), solving only for activation rates *ξ*_2_ and *ξ*_3_ by fitting to viral load. Simulations reproduce three phases of viral clearance (Fig. 6b) and predict trajectories of infected cell compartments (Fig. 6c). The model fit is flexible to assumptions of starting values of the three infected cell compartments (the relative proportion of which are unknown pre-ART): in this circumstance, we arrive at different values of *ξ*_2_ and *ξ*_3_ without impacting overall model conclusions regarding the HIV reservoir. The size of the sanctuary (expressed as the fraction of infected cells *φ*_S_) is only constrained to be below a value <10^−5^ to ensure accurate model fit for a static sanctuary model. This value can be higher for a decaying sanctuary (Fig. 7a, third column).

### Cellular proliferation sustains HIV infection during ART

We next used the model to estimate the fraction of cells generated by cellular proliferation versus viral replication. We conservatively assumed that prior to ART all infected cells were generated by viral replication. Then, we tracked the number of cells whose origin was replication and the number whose origin was cellular proliferation. Without directly simulating a phylogeny, the fraction of all cells that derive from replication provides a surrogate for the expected fraction of cells that would give a signal of evolution. We also distinguish the current replication percentage, the fraction of infected cells currently being generated from viral replication, from the net replication percentage, the fraction of total infected CD4+ T cells remaining at a given time whose origin was HIV replication. This distinction contrasts the surviving, historically infected cells with those presently being generated via HIV infection. Because many long-lived cells were once generated by HIV infection, the net replication percentage typically exceeds the current replication percentage.

We simulated the model under several plausible sanctuary and reservoir conditions to assess the relative contributions of infection and cellular proliferation in sustaining infected cells. We considered different reservoir compositions based on evidence that effector memory (T_em_), central memory (T_cm_), and naive (T_n_) cells proliferate at different rates and that distributions of infection in these cells differ among infected patients^12,42,43^. Further, because a drug sanctuary has not been observed, its true volume is unknown and may vary across persons. We therefore conducted simulations with a static sanctuary, a slowly diminishing sanctuary, and no drug sanctuary (Fig. 7a).

Regardless of assumed pre-treatment reservoir composition and sanctuary size, the contribution of replication to generation of new infected cells is negligible after one year of ART. The contribution of current replication diminishes rapidly with time on ART regardless of whether a sanctuary is assumed (Fig. 7b). The fraction of long-lived latently infected cells (*I*_3_) generated by viral replication (Fig. 7c, note log scale) is negligible within days of ART initiation. This finding captures the extent of the impact of proliferation even when a sanctuary is assumed.

### A fossil record of prior replication events

In all simulations, the net fraction of cells generated from viral replication rather than cellular proliferation at 6 months of ART (5–25% in Fig. 7d) is higher than the current percentage generated by replication (Fig. 7b). A higher fraction of slowly proliferating T_n_ cells exacerbates the difference between historical and contemporaneous generation of infected cells (Fig. 7d, green line). Because the net fraction is what will be observed experimentally, the model reveals why ongoing evolution might be observed even while the dominant mechanism sustaining the reservoir is cellular proliferation. In keeping with the first section of our paper, after 12 months of ART, the net and current percentage of infected cells generated by HIV replication become negligible for all simulated parameter sets. The lag between net and current viral replication generation emerges whether or not a small drug sanctuary is included in the model.

**Fig. 8:**
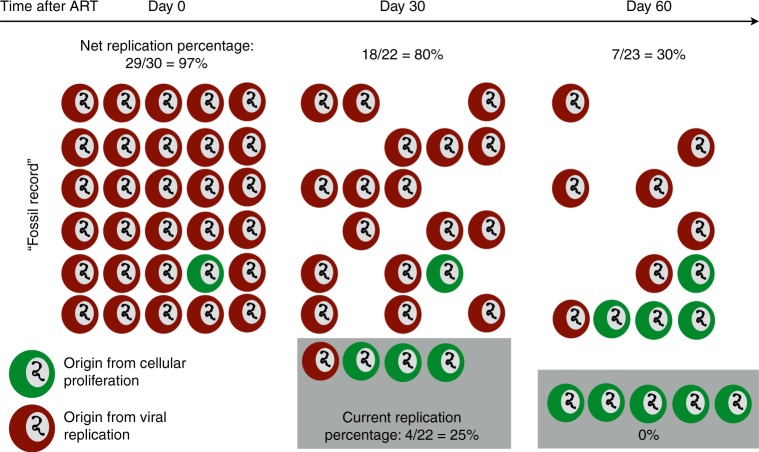
Qualitative illustration of the fossil record phenomenon. In an example population of 30 infected cells, the proportion of infected cells that were once generated by HIV replication (the net replication percentage, or fossil record of HIV replication) remains >30% for the first 2 months of ART. However, in this time, the proportion of cells newly generated by HIV replication (current, shaded box) becomes negligible. The net fraction is observed experimentally, so our simulations indicate a contemporaneous representation of the HIV reservoir cannot be observed until the fossil record is completely washed out, sometime between 6 months and a year of ART

We refer to the phenomenon that long-lived cells may contain signatures of past viral replication as the fossil record. To emphasize the concept, the fossil record finding is qualitatively illustrated in Fig. 8 using a population of 30 infected cells. At 3 time points following the initiation of ART, we compare the net and current percentage of cells generated by viral replication. At day 60, 30% of cells remain that were originally generated by viral replication. This means 30% of observed sequences might produce a signal of evolution. However, at that time an overwhelming majority of new infected cells are being generated by proliferation.

### Differing drivers of observed and current replication

**Fig. 9:**
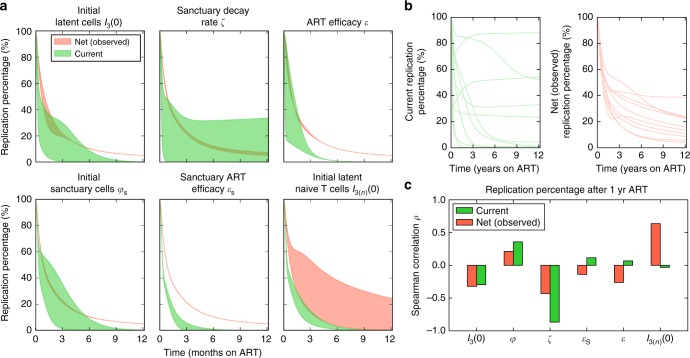
Sensitivity analysis of model results. **a**–**c** See Methods for complete simulated parameter ranges. **a** Local sensitivity analysis (green: current; red: net, or observed) revealed no meaningful difference in percentage of new infected cells generated by viral replication after a year of ART despite variability in initial reservoir volume *I*_3_(0), sanctuary fraction *φ*_S_, and ART effectiveness in and out of the sanctuary (${\it{\epsilon }}_{\mathrm{S}}$ and ${\it{\epsilon }}$). Only an extremely low, or zero, sanctuary decay rate *ζ* predicted that a meaningful percentage (25%) of infected cells would be newly generated by HIV replication at one year, despite the fact that signals of evolution are not typically observed at this time point. Including a high percentage of slowly proliferating naive CD4+ T cells (T_n_) in the reservoir alters the percentage of net, but not current, replication percentage. **b** 25 examples from 1000 global sensitivity analysis simulations. HIV replication accounted for fewer than 25% of current and net infected cells after a year of ART in a majority of simulations. **c** The parameters most correlated with current and net replication percentage at 1 year of ART are different. Current replication percentage inversely correlates with sanctuary decay rate while net (observed) replication percentage positively correlates with reservoir composition (quantified with the fraction of naive latently infected cells). Correlations are measured with the Spearman correlation coefficient

We next performed sensitivity analyses to identify parameters that impact the timing of transition from HIV replication to cellular proliferation as a source for current and observed infected cells. Under all parameter assumptions, the majority of current infected cells arose from proliferation after a year of chronic ART (Fig. 9a). Only the sanctuary decay rate (*ζ*) had an important impact on generation of current infected cells. When target cell availability did not decay at all, 25% of current infected cells were generated by HIV replication after a year of ART (Fig. 9a)—not consistent with lack of viral evolution observed at this time point. Rapid disappearance of HIV replication as a source of current infected cells was identified regardless of initial reservoir volume, drug sanctuary volume, ART efficacy, and reservoir composition (fraction of T_em_, T_cm_, and T_n_).

The net replication percentage was not affected by the decay rate of target cells within the drug sanctuary. Only an increase in the percentage of slowly proliferating reservoir cells (T_n_) predicted an increase in the net replication percentage (Fig. 9a). The drivers of current infected cell and net infected cell origin therefore differed completely, highlighting the major differences between observed sequence data and contemporaneous mechanisms generating new infected cells.

To confirm these results, we simulated 10^4^ patients in a global sensitivity analysis in which all parameter values were simultaneously varied. A rapid transition to proliferation as the source of new infected cells occurred during year one of ART in a majority of simulated patients, and the same variables correlated significantly with net and current replication percentage, respectively (Fig. 9b, c). Overall, this analysis does not rule out the possibility of a drug sanctuary but confirms that its impact relative to cellular proliferation is likely to be minimal.

## Discussion

To eliminate HIV-infected cells during prolonged ART, it is necessary to understand the mechanisms by which they persist. We use existing data and two methods—inference of HIV clone distributions and mechanistic mathematical modeling—to determine that a majority of infected cell persistence is due to cellular proliferation rather than HIV replication. Strategies that enhance ART delivery to anatomic drug sanctuaries are less likely to reduce infected cell burden relative to lymphocyte anti-proliferative therapies.

While the raw data indicate substantial fractions of observed singleton sequences, when the total reservoir size is considered, these observed singletons are revealed to be members of clonal populations. The HIV reservoir appears to be defined by a rank-abundance distribution of clone sizes that can be roughly approximated as a power-law relationship. This distribution implies that a small number of massive clones and a massive number of small clones together-comprise a large percentage of sequences.

A power-law distribution can be created when a heterogeneous population grows multiplicatively with a widely variable growth rate^55^, which suggests that the distribution of clone sizes in the reservoir has a mechanistic basis. It is plausible, though unproven, that variable growth arises from rapid bursts of CD4+ T cell proliferation due to cognate antigen recognition. HIV integration into tumor suppression genes could also account for clonal dominance^36,37^. Smaller clones may arise from homeostatic proliferation, or less frequent exposure to cognate antigen.

While we cannot rule out cellular longevity as a cause of HIV persistence in certain cells, the observation of multiple clonal sequences cannot arise purely from long-lived latently infected cells. Our analysis suggests that most observed singleton sequences arise from populations that have undergone many rounds of clonal proliferation.

We developed a mathematical model because our inference techniques do not capture time-dynamics of the reservoir or reconcile observations from early and late ART. This model is the first to include the three main mechanistic hypotheses for reservoir persistence: an ART sanctuary, long-lived latent cells, and latent cell proliferation. The model recapitulates known HIV RNA decay kinetics while tracking cells that originate from ongoing replication and cellular proliferation.

We demonstrate why a fossil record of evolution is observed early during ART, whether or not a small drug sanctuary exists. The model differentiates the fraction of infected cells contemporaneously generated by HIV replication (current replication percentage) from the fraction that were generated by viral replication in the past (net, or observed, replication percentage). The observed replication percentage remains non-negligible in the first months of ART while the current replication percentage drops rapidly. An observed sequence that gives a signal of divergence from the founder virus likely represents a historic rather than a current replication event. Because time of detection does not correlate linearly with sequence age, inference of evolution early during ART is problematic^20,21^. However, the fossil record is transient: within a year of effective ART, observed phylogenetic data represents true reservoir dynamics. Our model agrees with observations reflecting a lack of contemporaneous HIV evolution after this time^14,22–27,29,30,36,37^.

Our sensitivity analysis shows that the variable correlating with higher observed replication percentages (larger proportion of slowly proliferating CD4+ T cells in the reservoir) differs from the variable correlating with higher current replication percentages (slower decrease in sanctuary size). Yet, only current replication percentage represents the true amount of ongoing HIV evolution. Without requiring any phylogenetic simulation, this simple model provides an explanation for observed pseudo-evolution during the first months of ART and none thereafter^14,22–27,29,30,36,37^. If we assume a large drug sanctuary that does not contract with time, a persistent low-level sanctuary would emerge that stabilizes at 6 months and generates ongoing evolution at later ART time points. Notably, this has not been observed in clinical studies.

Our modeling results inform experiments in two ways. First, using rarefaction, we suggest sample sizes to verify our hypotheses experimentally (Supplementary Fig. 4). Observed values of sequence richness and clone size, are substantial underestimates. Current studies only sample the tip of the iceberg of the HIV reservoir. Hundreds of thousands of infected cells from a single time point would be required to capture true reservoir diversity. This sampling depth could only be feasibly achieved as part of an autopsy study. Second, we demonstrate that the wash-out period for the fossil record may be up to a year post ART. Future reservoir studies should be conducted after this time point to avoid observation of historic rather than contemporaneous evolution.

Our study has important caveats. Current integration site data, while robust, is limited to a handful of participants in only a few studies. Modeling rank-abundance curves makes a large assumption about the continuity of the data. The power-law model represents but one approach. Future work should address why that distribution provides good fit to the data. Extrapolating abundance curves has been criticized: our attempt to design a simple parametric model was based on the additional information of reservoir size and our goal to define an upper limit on reservoir richness^56^; nevertheless, the tail of our distributions is impossible to precisely characterize with our methods.

Our approach is calibrated against sequence data from blood. However, the dynamics of HIV within lymph tissue may have different distributions. While historically, blood samples have been taken as a surrogate for HIV-infected cells, we cannot rule out a small drug sanctuary that does not exchange virus or infected cells with blood. It seems unlikely that such a sanctuary could be sustained because some trafficking of CD4+ T cells from other compartments may be necessary to avoid terminal target cell limitation.

In conclusion, we demonstrate that the majority of HIV-infected cells arise from proliferation during ART and provide an explanation for incongruent observations of evolution before and after a year of ART. Because proliferation is the dominant force sustaining the HIV reservoir^34^, we suggest limiting proliferation as a prime therapeutic target^10,11,57^.

## Methods

### Rank-abundance of HIV integration sites

We used an ecological framework to study the abundance of clonal HIV. To do so, we applied methods to integration site and replication-competent HIV sequence data. Unique sequences or integration define distinct clonal populations. The population size of that clone defines its abundance. By ranking the clones from largest to smallest by abundance, we developed a rank-abundance curve, *a*(*r*), for each participant time point. No assumptions were made about the stability or dynamics of the reservoir rank-abundance over time.

In our analysis of data from Wagner et al.^37^, we combine measurements taken closely in time and use the median time point as in the original publication. In our analysis of Maldarelli et al.^36^, we used unedited published integration site counts. It is important to note that the methods used by Wagner et al. and Maldarelli et al. are slightly different. The ISLA method used by Wagner et al. is lower throughput than the next generation shotgun sequencing method used by Maldarelli et al. The absolute number of viruses identified by each group therefore differs. However, the fraction of observed singletons is similar between the two studies. We manually counted the abundance of replication-competent HIV sequences from phylogenetic trees in Hosmane et al.^34^.

### Calculation of rarefaction curves

We used rarefaction curves to estimate the expected number of distinct sequences that would still be present in a subsample of *k* sequences from the observed data with sample size of *N*:

1 $$\left\langle {n_k} \right\rangle = R^{{\mathrm{obs}}} - \left( {\begin{array}{*{20}{c}} N \\ k \end{array}} \right)^{ - 1}\mathop {\sum }\nolimits_{r = 1}^{R^{{\mathrm{obs}}}} \left( {\begin{array}{*{20}{c}} {N - a(r)} \\ k \end{array}} \right),$$

where the parentheses indicate binomial coefficients, e.g., $({{N}\atop {k}}) = \frac{{N!}}{{k!\left( {N - k} \right)!}}$. Later, we extrapolated rarefaction curves using the modeled distributions for the total reservoir size *L*. Because the number of samples we allowed was orders of magnitude smaller than the number of cells in the reservoir, *k* ≪ *L*, we used Stirling’s approximation to simplify the binomial coefficients. The expected number of sequences after *k* samples is then

2 $$\left\langle {\tilde n_k} \right\rangle = R - \mathop {\sum }\nolimits_{r = 1}^R \left[ {1 - \frac{{a(r)}}{L}} \right]^k,$$

an expression which avoids computation of large factorials (derivation in the Supplementary Methods).

### Nonparametric estimation of species richness

We employed the Chao1 estimator to set a lower bound on the sequence or integration site richness^58^. A derivation of the estimator is included in the Supplementary Methods. Chao1 is not a mechanistic model and requires no free parameters. Inference relies on only the number of observed singleton (*N*_1_) and observed doubleton (*N*_2_) sequences such that

3 $$R^{{\mathrm{Chao1}}} = R^{{\mathrm{obs}}} + \frac{{N_1(N_1 - 1)}}{{2(N_2 + 1)}}.$$

We display an asymmetric confidence interval in Fig. 3 (see Chao et al.^58^ or Supplementary Methods for the definition). We also note it is possible the data are undersampled to the extent that a one-sided confidence interval may be more appropriate. Thus, for our biological conclusions we take the Chao1 point estimate as a lower bound, and constrain the upper bound using the parametric model (Eq. 4). Other richness estimators (jackknife 1 and 2) were tested but provided similar and consistently lower estimates of richness than the Chao1 estimator. These were not included in our results because the Chao1 was interpreted as a lower bound on true sequence richness.

### Parametric models of rank-abundance

Estimates of the size of the HIV reservoir (both replication-competent and total) were gathered from the published literature^33^. We then developed a parametric model to quantify the true rank-abundance distribution of the complete HIV reservoir. Examination of the data indicated a possible log-log-linear relationship, so we chose a discrete integer power-law model so that the probability of a rank is described by *p*(*r*) = *ψ*(*R*)*r*^−*α*^ where the coefficient $\psi \left( R \right) = \mathop {\sum}\nolimits_{r = 1}^R {r^{ - \alpha }}$ is the normalization constant for the power-law. Then, to describe the true rank-abundance *a*(*r*) we chose the reservoir size depending on the model context (replication-competent *L* = 10^7^ or total HIV DNA *L* = 10^9^). To ensure integer number of cells, we rounded this distribution, and forced the total number of cells to equate with reservoir size. That is,

4 $$a\left( {r;\alpha ,R,L} \right) = \left| {\left[ {L\psi (R)r^{ - \alpha }} \right]} \right|$$

where |[]| indicates rounding to the nearest integer. Thus, our model depended on two free parameters, a power-law exponent *α*, and the reservoir richness *R*. Other functional forms were explored but simplicity and accurate reproduction of the data were optimal with the power-law.

### Fitting rank-abundance models

Using the experimental data we found the best-fit model using the following procedure. We fixed the reservoir size *L* depending on the model context (replication-competent or total HIV DNA). We chose a value for *R* and *α* from ranges *R* ∈ [10^3^,10^7^] and *α* ∈ [0,2] to specify the model. Then, we sampled the extrapolated distribution 10 times using multinomial sampling with the same number of samples as the experimental data being fit, ${\cal M}(N,p(r))$. This procedure assumes that sampling cells does not change the distribution of the reservoir, which is reasonable given the reservoir size. Each sampled data set was compared to the experimental data by computing the residual sum of squares (rss) error of the cumulative proportional abundance (cpa) curves. For each model then, the reported error is the average rss over the 10 resamplings. Because the rss error is not symmetric across the domain of the cpa, this approach becomes similar to minimizing the Kolmogorov–Smirnov (KS) statistic: the maximum deviation between two cumulative distributions. For each experimental data set 2500 model parameter sets were generated, and fitting results were visualized as heat maps (see Figs. 4a and 5a for example). Because the procedure becomes computationally expensive as *R* > 10^7^, we did not explore values above this threshold. In theory, it is possible to have a distribution with all clones having a single member *R* = *L*, *α* = 0. For the total DNA reservoir, this value would result in *R* = 10^9^. However, this model was never optimal. In fact, as richness increased beyond ≈10^6^, the model was no longer sensitive to *R*. Thus, it appeared that finding the best-fit *α* was sufficient to specify the model if proper bounds on richness were included.

We excluded models where *R* < *R*^Chao1^, but we also sought to identify an upper bound for *R*. Indeed, certain model parameter combinations are mathematically impossible. For example, for a given power-law exponent, the richness is constrained below a certain value for a given reservoir size. Similar arguments have been made in ecology under the terminology of feasible sets^59^. To determine the largest possible richness that still optimized fit, we chose the roughly constant value of *α* that emerged when *R* was large enough to be unidentifiable. Then, we noted that for large *R* it is reasonable to allow $\mathop {\sum}\nolimits_{r = 1}^R a (r) = {\int}_1^R a (r){\mathrm{d}}r$. *R* is thus approximately bounded, and we solved for the maximal value or the upper bound on the richness given the best-fit *α* and the chosen *L*. A discussion and numerical validation of this approximation is presented in the Supplementary Methods and Supplementary Fig. 2. Choosing the model with largest richness provides the sequence abundance most permissive of true singleton sequences—the model most favoring ongoing replication as an explanation for HIV persistence. In extrapolated reservoirs, we used the maximum richness model to ensure we were biasing the results as strongly as possible against our own hypothesis.

### Model fitting validation with simulated data

A discussion and demonstration of model validation is included in the Supplementary Methods and Supplementary Fig. 1. The exercise shows that simply fitting a power-law to the experimental data (using log-log-linear regression) without the extra sampling step necessarily underestimates the power-law exponent, demonstrating the utility of our approach. Moreover, it shows that a published maximum likelihood approach^60^ is not as accurate for these data as our resampling approach (code hosted at http://tuvalu.santafe.edu/~aaronc/powerlaws/ last accessed July 2018). We simulated a reservoir with known power-law exponent Supplementary Fig. 1A and tested for recovery of this known value. The fitting validation proceeded identically to the data fitting: 2500 distributions were generated (225 examples are shown in Supplementary Fig. 1D), the simulated data was sampled Supplementary Fig. 1B, and reranked Supplementary Fig. 1C. Fitting results Supplementary Fig. 1E, F are shown analogous to Figs. 4 & 5A, B. Model comparison demonstrating optimal accuracy with our resampling approach is shown in Supplementary Fig. 1G.

### Mechanistic model for the persistence of the HIV reservoir

The canonical model for HIV dynamics describes the time-evolution of the concentrations of susceptible *S* and infected *I* CD4+ T cells and HIV virus *V*^50,54,61^. Our model grows from the canonical model, simplifying with several approximations and extending the biological detail to simulate HIV dynamics on ART, including a long-lived latent reservoir and a potential drug sanctuary. Perelson et al. first noticed and quantified a biphasic clearance of HIV virus upon initiation of ART and showed that viral half-lives of 1.5 and 14 days correspond with the half-lives of two infected cell compartments^50,54^. With longer observation times and single-copy viral assays, Palmer et al.^51^ documented four-phases of viral clearance after initiation of ART. Because of uncertainty in distinguishing the third and fourth phase in that study, we focus on the first three decay rates and corresponding cellular compartments, attributing a mixture of the third and fourth phase decay to the clearance of the productively infectious latent reservoir (half-life 44 months) as measured by Siliciano et al.^3^ and corroborated by Crooks et al.^2^ and the clearance of HIV DNA^47^. We developed a mechanistic mathematical model that has three types of infected cells *I*_1_, *I*_2_, *I*_3_ that are meant to simulate productively infected cells, pre-integration infected cells, and latently infected cells, respectively. We classify rapid death *δ*_1_ and viral production within actively infected cells *I*_1_. Cells with longer half-life that may represent pre-integration infected cells *I*_2_ are activated to *I*_1_ at rate *ξ*_2_. *I*_2_ may represent CD4+ T cells with a prolonged pre-integration phase, but their precise biology does not affect model outcomes^48^.

The state *I*_3(*j*)_ represents latently infected reservoir cells of phenotype *j*, which contain a single chromosomally integrated HIV DNA provirus^44^. *I*_3_ reactivates to *I*_1_ at rate *ξ*_3_ which at present is assumed to be constant across cell phenotypes^49^. The probabilities of a newly infected cell entering *I*_1_, *I*_2_, *I*_3(*j*)_, are *τ*_1_, *τ*_2_, *τ*_3(*j*)_. Because we are focused on the role of proliferation, we assume sub-populations of *I*_3_^12^, including effector memory (T_em_), central memory (T_cm_), and naive (T_n_) CD4+ T cells, which proliferate and die at different rates *α*_3(*j*)_, *δ*_3(*j*)_^12,42,43^. Parameter values and initial conditions for the model are collected in Table 1.

### Modeling with an ART sanctuary

A recent hypothesis about reservoir persistence suggests there may be a small, anatomic sanctuary (1 in 10^5^ infected cells) in which ART is not therapeutic^4^. Thus, we included the state variable *I*_S_ that is maintained at a constant set-point level prior to ART, where all new infected cells arise from ongoing replication. The amount of virus produced by the sanctuary *V*_S_ is extremely low relative to non-sanctuary regions because ART results in levels undetectable by sensitive assays^51^.

Many studies have demonstrated that HIV accelerates immunosenescene through abnormal activation of CD4+ T cells^62–64^. ART results in a marked reduction of T cell activation and apoptosis, a potential signature of HIV susceptible cells^65^. By examining the decline of activation markers for CD4+ T cells, we approximated the decay kinetics of activated T cells upon ART, inferring approximate decay kinetics of the target cells in our model^52,53,66^. A range of initial values exists (from ~5 to 20% activation) depending on stage of HIV infection, yet after a year of ART, a large percentage of patients return to almost normal, or slightly elevated CD4+ T cell activation levels (2–3%)^52^. Because we assume that target cell depletion is minimal at viral load set-point, we allow susceptible cell concentrations to decrease over time as immune activation decreases. We choose an exponential model, i.e., *S* = *S*(0)e^−*ζt*^, which is an obvious simplification (it could also be biphasic but the data are not granular enough to discriminate this dynamic subtlety). From existing data, the decay constant should be in the range *ζ* ~ [0.002, 0.01] day^–1^ ^52,66^. We extend this decay into the sanctuary, allowing the number of susceptible cells over the whole body to decrease so that *I*_S_ = *I*_1_(0)*φ*_S_e^−*ζt*^ where *φ*_S_ is the fraction of infected cells in a sanctuary. Model simulations are also performed without this assumption of target cell contraction.

Last, we use the quasi-static approximation that virus is proportional to the number of actively infected cells in all compartments *V* = *n*(*I*_1_ + *I*_S_) where *n* = *π*/*γ*, the ratio of the viral production rate to the viral clearance rate (Table 1). The model is thus

5 $$\begin{array}{l}\dot I_1 = \tau _1{{\beta }}_{\it{\epsilon }}{{SV}} - \delta _1I_1 + \xi _2I_2 + \mathop {\sum }\nolimits_j \xi _3I_{3(j)}\\ \dot I_2 = \tau _2{{\beta }}_{\it{\epsilon }}{{SV}} + \left( {\alpha _2 - \delta _2 - \xi _2} \right)I_2\\ \dot I_{3(j)} = \tau _{3(j)}{{\beta }}_{\it{\epsilon }}{{SV}} + \left( {\alpha _{3(j)} - \delta _{3(j)} - \xi _3} \right)I_{3(j)},\end{array}$$

where the over-dot denotes derivative in time.

### Comparing proliferation to viral replication

By solving the ODE model (Eq. 5), we computed the total number of newly infected cells generated in a given time interval Δ*t* by ongoing replication. That value is $I^{{\mathrm{rep}}}(t) = \left( {\beta _{\it{\epsilon }}{{SV}} + \phi _{\mathrm{S}}\beta {{SV}}_{\mathrm{S}}} \right){\mathrm{\Delta }}t$. The total number of newly infected cells generated by proliferation of a previously infected cell can be computed similarly in a time interval as $I^{{\mathrm{pro}}}(t) = \mathop {\sum}\nolimits_{i(j)} {\alpha _{i(j)}I_{i(j)}{\mathrm{\Delta }}t}$. Therefore, the percentage of infected cells generated by current replication is written

6 $${\mathrm{\Phi }}^{{\mathrm{current}}}\left( t \right) = 100 \cdot \frac{{I^{{\mathrm{rep}}}(t)}}{{I^{{\mathrm{rep}}}(t) + I^{{\mathrm{pro}}}(t)}}.$$

We can further subset this current replication fraction by examining the percentage of infected cells that enter the long-lived latent state *I*_3_ by defining $I^{{\mathrm{rep}}(3)}(t) = \tau _3(\beta _{\it{\epsilon }}{{SV}} + \phi _{\mathrm{S}}\beta {{SV}}_{\mathrm{S}}){\mathrm{\Delta }}t$ and $I^{{\mathrm{pro}}(3)}(t) = \mathop {\sum}\nolimits_j {\alpha _{3(j)}I_{3(j)}{\mathrm{\Delta }}t}$ so that

7 $${\mathrm{\Phi }}^{{\mathrm{current}}(3)}\left( t \right) = 100 \cdot \frac{{I^{{\mathrm{rep}}(3)}(t)}}{{I^{{\mathrm{rep}}(3)}(t) + I^{{\mathrm{pro}}(3)}(t)}}.$$

The net (or observed) replication percentage, is the fraction of cells that were once generated by viral replication. To compute this quantity, we use an additional set of ODEs that we refer to as tracking equations because they do not change the dynamics of the system, and only are used to track specific variables. To denote the net value as opposed to current value we use a superscript Σ. The net cells generated by viral replication in state *i* of phenotype *j* is governed by the differential equation

8 $$\dot I_{i(j)}^{({\mathrm{\Sigma }}){\mathrm{rep}}} = \tau _{i(j)}{{\beta }}_{\it{\epsilon }}{{SV}} - \left( {\delta _{i(j)} - \xi _{i(j)}} \right)I_{i(j)}^{({\mathrm{\Sigma }}){\mathrm{rep}}}.$$

Likewise, the net cells generated by proliferation in state *i* of phenotype *j* is governed by the differential equation

9 $$\dot I_{i(j)}^{({\mathrm{\Sigma }}){\mathrm{pro}}} = \alpha _{i(j)}I_{i(j)} - \left( {\delta _{i(j)} - \xi _{i(j)}} \right)I_{i(j)}^{({\mathrm{\Sigma }}){\mathrm{pro}}}.$$

We note that because we only allow these two mechanisms, $\dot I_{i(j)} = \dot I_{i(j)}^{({\mathrm{\Sigma }}){\mathrm{rep}}} + \dot I_{i(j)}^{({\mathrm{\Sigma }}){\mathrm{pro}}}$ and $I_{i\left( j \right)}(t) = I_{i\left( j \right)}^{\left( {\mathrm{\Sigma }} \right){\mathrm{rep}}}(t) + I_{i\left( j \right)}^{({\mathrm{\Sigma }}){\mathrm{pro}}}$. We solved the tracking equations separately and took the sum over cell types and phenotypes. Ultimately, the net replication fraction is

10 $${\mathrm{\Phi }}^{\mathrm{\Sigma }}\left( t \right) = 100 \cdot \frac{{\mathop {\sum }\nolimits_{i(j)} I_{i(j)}^{({\mathrm{\Sigma }}){\mathrm{rep}}}(t)}}{{\mathop {\sum }\nolimits_{i(j)} I_{i(j)}^{({\mathrm{\Sigma }}){\mathrm{rep}}}(t) + I_{i(j)}^{({\mathrm{\Sigma }}){\mathrm{pro}}}(t)}}.$$

In all simulations, we assumed that 100% of infected cells at the initiation of ART were generated by viral replication, that is Φ^Σ^(0) = 100. This assumption biases results in favor of replication. However, we choose it because, to the best of our knowledge, studies of proliferation during chronic untreated HIV have not been performed.

### Sensitivity analysis

Using estimated parameter bounds [lower, upper], we completed a local and global sensitivity analysis. These ranges were chosen to cover a wide range of possible assumptions. We allowed *I*_3_(0) = [0.02,2] cells µL^−1^, *φ*_S_ = [10^−6^,10^−4^] unitless, *ζ* = [0,0.2] day^−1^, ${\it{\epsilon }} = [0.9,0.99]$ unitless, ${\it{\epsilon }}_{\mathrm{S}} = [0,0.9]$ unitless, *I*_3(*n*)_(0) = [0,0.5] × *I*_3_(0) cells µL^−1^. For the local analysis, we used all values as in Table 1 and modified one parameter at a time over each listed range above. The global analysis was performed in Python by using 10^4^ Latin Hypercube samplings of the complete 6-dimensional parameter space using PyDOE^67^. The key outcome, the replication percentage (net and current) at 1 year of ART, was correlated to each parameter using the Spearman correlation coefficient—defined by the ratio of the covariance between the outcome and the variable divided by the standard deviations of each when the variables were rank-ordered by value.

### Code availability

Computational code for all calculations and simulations was performed in Python and Matlab. and is freely available at https://github.com/dbrvs/reservoir_persistence.

## Electronic supplementary material

Peer Review File

Supplementary Information

## Data Availability

Sequence data were obtained from the Retrovirus Integration Database (RID)^68^. The authors declare that all other data supporting the findings of this study are available within the article and its Supplementary Information files, or are available from the authors upon request.
