# Supplementary material for: A majority of HIV persistence during antiretroviral therapy is due to infected cell proliferation
Source: Nat Commun. 2018 Nov 16;9:4811. doi: 10.1038/s41467-018-06843-5 (PMC6240116; doi:10.1038/s41467-018-06843-5)
Supplement: Supplementary file 2 — Supplementary Information [file 41467_2018_6843_MOESM2_ESM.pdf]

Reviewers' comments:

Reviewer #1 (Remarks to the Author):

* General remarks
To distinguish between rivaling hypotheses (replication vs proliferation) for the nature of the HIV-1 reservoir under ART, Reeves et al analyze with computational methods data from other, experimental studies (Wagner et al, Maldarelli et al, etc.). These experimental studies lend support to proliferation of infected cells as major reservoir of HIV-1 diversity.

First, Reeves et al characterize the diversity of HIV-1 in the patients from the studies mentioned above using mostly standard ecological methods (Chao index, rarefaction, rank abundance curves, power-law fits). The use of ecological methods to characterize intra-individual diversity of HIV-1 is legitimate and not new (e.g. Yin et al 2012 Retrovirology 9:108, Gregori et al 2014 Bioinformatics 30:1104). The authors find that the population is dominated by clonal HIV-1 sequences, probably resulting from proliferation of infected cells. This finding is consistent with the conclusions of the experimental articles mentioned above from which the authors have taken their input data.

Secondly, to explain the discordance between apparent evolution and dominantly clonal population, Reeves et al develop a system of ordinary differential equations to model the intra-individual dynamics of infection between several compartments (actively replicating infected cells, proliferating infected cells, etc.). The takeaway of this part is that under ART the contribution of active replication and therefore evolution to observed diversity should be small if not negligible, and that apparent evolution "represents a fossil record of prior replication events".

* Main concerns

I have the following main concerns:

1. The authors state early on (line 75) the importance of studying viral evolution to distinguish between competing models (ongoing replication vs proliferation) for the HIV-1 reservoir. I agree with this statement: to test the models we have to study viral evolution, which encompasses sequence *and* population dynamics over time. For instance, Lorenzo-Redondo et al 2016 Nature 530:51 (reference 4 of Reeves et al) show that observed HIV-1 sequences can be most easily explained by including ongoing evolution in the model. The clear molecular clock signal reported by reference 4 is more difficult to explain by "fossil" sequences. Estimating diversity, important as it is, covers only one aspect of the system, so that, in the end, the first part of the manuscript is not more conclusive than the articles from which the authors have acquired their experimental data.

2. I am not sure how trustworthy the results in the first part (diversity estimation) are. There are a number of errors in the formulas and open questions that draw into doubt the validity of the computational procedures, for instance:
a. line 692: I am pretty sure that the binomial coefficient of x over y cannot be reasonably

approximated by x^y (except for the trivial case of y=1); if the authors have used this approximation, their results are flawed
b. line 711: I am also quite sure that the formula for sigma^2 is wrong; there should be no R^obs
c. line 703ff: Use of Chao1 estimate: have the authors made sure that the conditions for application of Eq (3) are fulfilled (esp. homogeneity, see e.g. Chiu et al 2014 Biometrics 70:671)? Otherwise they systematically underestimate species richness. For instance the large variance in clone size could speak against homogeneity (lines 616ff).
The authors should carefully check formulas for applicability and correctness. They should either derive formulas or cite the corresponding literature.

* Minor concerns

1. Lines 154-161: The authors should make sure and show that the respective experimental protocols of Wagner et al and Maldarelli et al allow that their data can be directly compared (as the authors do in Fig 2A).

2. Fig 2, line 208: "The proportion of observed singleton HIV DNA sequences generally decreases with time on ART" (Fig 2A)
a. Fig 2A: Binomial 95% confidence interval error bars should be shown on the points to demonstrate that suspected weak trend is likely real.
b. Fig 2A: With 5 out of 6 individuals having (weakly) decreasing singleton fractions the 95% confidence interval for decreasing singleton fractions is [0.359,0.996] based on a binomial estimate, i.e. the probability for decrease of 0.5 still is comfortably included in the 95% confidence interval.
c. Fig 2E and 2F and description: "In both data sets, the morphology of observed rank abundance $a(r)$ was similar across samples (Fig 2E, F), suggesting a generic structure to clonal distribution of reservoir cells." How do the authors judge the "morphology"? Most rank abundance curves are hollow curves and thus trivially have a similar morphology. It is well possible that different cases (e.g. infected individuals but not under ART) also have "similar morphology" -- the authors should demonstrate that this is not the case, otherwise their argument of "similar morphology" is none.

3. Fig 3 and underlying formulas and descriptions
a. By "A bias-corrected estimator (avoiding division by zero)" the authors seem to imply that the purpose is to avoid division by zero. However, the modification of the Chao1 formula reduces the systematic deviation (=bias) between the richness estimate and the true richness (see Chiu et al 2014 Biometrics 70:671 and references therein). The avoidance of division by zero is a side-effect that could be achieved by other variants of Chao1, e.g. R^obs + f1^2/(2*f2+1), without changing the bias perceptibly.
b. Line 239: What is the lower bound estimate for *observed* sequence richness?

4. Lines 815-817: In their system of differential equations the authors allow changes between I2 and I3 only through activation (via I1). On the other hand they state (line 405) "I2 may represent CD4+ T cells with a prolonged pre-integration phase, but their precise biology does not affect model outcomes." So, why can there be no direct link between I2

and I3? What would be the effect of such a link on the dynamics?

5. 75/76: Better: Due to the high error rate in HIV reverse transcription ...

6. 77-79: Inaccurate statement on mutations that should be corrected: Both the synonymous and the non-synonymous mutations are generated by error-prone replication. Some of the non-synonymous mutations are then selected by host immunity.

7. 234: It is unclear why authors cite reference 41.

8. Last tick mark in Fig 2B is 2, should probably be 25.

9. Occasionally unusual wording that does not contribute to clarity, e.g.
a. 404: "we classify rapid death"
b. 668: "we developed the rank abundance curve" -> computed
c. 679: "we developed rarefaction curves" -> computed
d. 688/689/692: the "combinatorial factors" are generally known as *binomial coefficients*
e. 811: "virus is proportional" -> viral load
f. 828: "newly generated cells due to replication"

10. 668: The rank abundance curve is not "r(a)", but a(r).

11. 686: missing reference or derivation

12. 688: ambiguous formula; should probably read x!/y!/(x-y)!

13. 692: missing reference or derivation

14. 695: missing reference or derivation

15. 606: no comma

Reviewer #2 (Remarks to the Author):

Reeves et al. analyzed data from several papers (Maldarelli, et al, Wagner, et al., and Hosmane, et al.) to determine the likelihood that the HIV-1 reservoir is maintained by ongoing viral replication in addition to clonal proliferation. They use the estimated size of the clones and the number of "singletons" from the published integration sites data to calculate the probability that any of the infected cells persisting on ART are not clonal. They conclude that that virtually all infected cells are from proliferating populations of cells. They also developed a model that allows for viral replication in ART sanctuary sites and demonstrate that, despite this, cellular proliferation will comprise the reservoir within a few months after ART initiation. This detailed analysis of published data will be an important contribution to our understanding of the HIV reservoir. I have just a few comments that I

believe will improve the manuscript.

1) The exact nature of the long tail of HIV singletons is unknown and unknowable with current technology. They could say that their modeling is consistent with "all clones" but they should not say that they show that there are only clones. This would soften the conclusions which I think more appropriately fits the data.
2) It is not clear what is meant by the sentence in lines 146-147, "Yet, equivalent replication competent sequences are also found and at comparable frequencies." It sounds as if they are saying that intact proviruses are found in comparable frequencies as defective proviruses which is not the case. This sentence should be reworded.
3) All of the integration sites data available in the RID were obtained from blood. It is possible that the size and frequencies of clones in tissues is different. This limitation of the analysis should be addressed.
4) Although it may not affect the outcome model, the statement that a minority of proviruses are latently infected in pre-ART is not correct for patients who initiate ART in chronic infection. Latently infected cells accumulate on ART and are the majority after years of infection even prior to starting treatment. It is more likely that the cells that die in the first months include those that are readily reactivated. Those that were not latent pre-ART likely die within days of initiating ART.
5) Current integration sites data is still limited and, as a result, some of the conclusions are stated a bit strongly and should be softened. However, it should be more strongly stated that these results are in direct opposition to the Lorenzo-Redondo, et al. conclusions that the reservoir is sustained by ongoing cycles of viral replication.

Reviewer #3 (Remarks to the Author):

Overall:

HIV persists despite long-term antiretroviral therapy, and the mechanism for this persistence continues to be debated. The major proposed mechanisms are a) ongoing replication of virus, despite drugs, in a "sanctuary" site in the body, b) long lifespans of latently infected memory CD4+ T cells, c) proliferation - without virus production and cell death - of these latently infected cells. In this paper the authors use two distinct methods to suggest that (c) is the dominant mechanism. The first part uses an analysis of the clonal composition of cells in the latent reservoir - based on uniquely identifying the virus infecting them and showing that many latent cells are descendants of the same infection event - to argue that latent cell proliferation must be maintaining the reservoir during ART. The second part is a mathematical model of both ongoing HIV replication in a sanctuary and HIV persistence in latent cells, and puts limits on how much ongoing replication could contribute to the observed # of latently infected cells.

Altogether, I think both of the authors arguments are strong and that their unique approach is an important contribution to this debate, which is critical to deciding the future of HIV therapeutics. The authors inform their models with data wherever possible and consider a

range of possible scenarios and parameter values to evaluate the robustness of their conclusions. Overall their conclusions agree with those of many other papers (which they reference), but they have arrived at them by novel means, leading further credence to them.

I have a few suggestions for how the authors could improve this work. As I outline below, there are multiple areas where explanations of the models and results are confusing and could be improved. More importantly, I think the authors need to do a better job of providing a coherent transition between the two very separate analyses in their paper (statistical analysis of clonality of reservoir from genetic data and mathematical model of ongoing replication vs latent cell dynamics). There isn't really any connection between these two halves of the paper, except that they are both related to the mechanism of HIV persistence during ART. In addition, they authors could do a better job - at minimum in the Discussion - of acknowledging the major assumptions and hence limitations of their work. The conclusion of the first half of the paper is almost entirely based on assuming that the clone size distributions follow a particular model and extrapolating this model out to the entire reservoir composition. Of course there are many reasons why this model may not be correct. The conclusion of the second half is based on the assumption that target cell availability in a proposed sanctuary actually declines during ART, an idea which has so far not been including in any other models of HIV dynamics and is hard to directly provide experimental evidence for, since no one really knows which subset of CD4+ T cells is actually susceptible to HIV (only a very small fraction of cells are ever infected even during peak viremia).

I think that the authors can easily make the suggested changes to the paper and that it will be a very appreciated contribution to this important debate in the HIV field.

Methods:

~668: Should explain that "abundance" in rank-abundance curves refers specifically to the relative proportion of the population composed of a particular clone
~686: You haven't yet explained what capital R stands for

~670: Thoughout the paper, but especially here, the way in which the distribution of clone sizes is manipulated to generate these derivative distributions such as "rank abundance" and "rarefaction curves" is quite confusing and the relevant terms are not defined clearly. For example, what is Robs? I thought it was the number of distinct clones observed in a sample. But, in line 684, you describe in words a quantity that seems exactly to be R_obs, and then give a formula for it, but this formula involves R_obs! So what is the thing you are defining in this line vs what is R_obs? Similar point for Equation 2. And what is the difference between k and N? Which is the sample size and what is the total population size? Both are referred to as sample sizes in the text. And what is Nobs and how does it differ from N? Is this formula referring to resampling the sample or sampling the population? Something cannot be an equation without having an equal sign with something on both sides. So what quantity is being defined by each of these formulas? I think the left hand side should say "R_obs(k) =" and the right hand side should be your formula but "R_obs"

should be replaced by "R_obs(N_obs)".

I think it is worth pointing out that a "rank abundance curve" is actually just a probability distribution/histogram - all the quantities add up to 1. I think "rank abundance distribution" would be a better name than "rank abundance curve" for this reason.

~692: This approximation for the binomial coefficients is not correct. See https://math.stackexchange.com/questions/1447296/stirlings-approximation-for-binomial-coefficient for correct formula.

Re Chao estimator: It would be nice to have a bit of an explanation for what is the theoretical basis for this estimator, and why you can claim it is "validated".

~736: The method described for fitting the proposed log distribution to the observed rank abundance distribution is not very statistically rigorous. Sum of squared errors approaches are not appropriate for CDF as they are more heavily weighted by values far from 0 and 1, and your current method is limited by the increments of parameter values you choose and the number of sample draws you simulate. Also, it does not properly take into account the sampling process. A more formally correct way would be to write down a likelihood function for a given observed set of data under give model parameters, and then either use formal optimization techniques to maximize the likelihood, or, use a Bayesian approach and sample from the posterior using MCMC to generate an estimate - with credible intervals - for each parameter value.

Line 789 - I think the analysis in Luo et al (http://rsif.royalsocietypublishing.org/content/10/84/20130186) is much more trustworthy source of estimates for the size of a compartment for residual replication, given that Lorenzo-Redondo et al is based on faulty estimates of reservoir evolution when in fact they were not measuring the long-lived reservoir at all.

Equation 6: This model is extremely poorly explained here. It is described very clearly in the main text, but then if someone is just reading the methods, there is no explanation. It would be good to either refer readers back to the main text, or repeat some of it here. More generally, the "Results" and the "Methods" section each contain a random subsample of the methods - might be more coherent to keep them in one place.

~821: These is something grammatically wrong with the first (non-bolded) sentence that makes it nonsensical. What does this formula mean? What are the numerator and denominator? Since you haven't mentioned anything about proliferation yet it is totally unclear.

Eq 9 - 11 : Do not understand what is going on here.

~853: Explain what these parameters are and what these ranges mean (where did they come from, what units are they in, etc).

Introduction:

~75-78. There are a few misleading statements about evolution here. Firstly the sentence that starts with "Due to .." does not really make sense. The factors mentioned at the beginning of the sentence are not why evolution implies replication. Evolution implies replication because we assume that the only way in which the genetic composition of a population can change is by mutation and selection, and we assume both require replication and thus turnover of a population. Note that this is not actually true though … there are other reasons why genetic composition could change … e.g. migration, differential death rates, etc. In regards to the next sentence, the high mutation rate of HIV RT is what is responsible for generating a huge amount of diversity on which selection can act. Immunological "pressure" is what actually facilitates selection. The large population size means that selection can be efficient, when compared to drift. For both synonymous and non-synonymous mutations, the mutations themselves are generated by error prone replication. The latter can be subject to selection.

~84: I think that the reference to 18 here is incorrect. I don't think Rosenbloom et al commented on undersampling .. their point was about differential decay of infected cells (exactly as you've included in your later model) masquerading as evolution during early ART initiation.

Results:

The heading of the section starting at 197, and the title for the caption of Figure 3, seem to be overstatements. You do not actually have proof of either of these statements. They are simply predictions of your model.

The Chao estimator should probably be described briefly here - what assumptions does it make and what theory is it based on? How does it make a prediction for the total species richness?

Line 269 - 278 : This fitting method is already described in the Methods and doesn't have to be mentioned in such detail here. Just say that you fit the observed distribution to a log-linear function to determine the best-fit parameters.

Figure 4: The difference between panels C/D vs E/F is not clear from the caption. Same for Figure 5.

~404: It's not clear to me why you decided to include in your model the complicating factor that target cells decline during ART. It seems like this is biasing towards your hypothesis that ongoing replication is not important. Why bother including this? And why bother including the multiple subtypes of latently infected cells?

Figure 7: I don't understand what "net replication percentage" is

In line 458 you mention a sanctuary size of 0.1% (e.g. 1 in 10^3), but then in Figure 7 you

only show much smaller sanctuary sizes - 1 in 10^5 and 1 in 10^6. Why? Wouldn't you conclusions about relative % of cells from new replication change if you used larger sanctuary sizes? Isn't the sanctuary size constrained by the total amount of residual viremia observed during ART? (around 1 copy/mL, which is ~10^4 lower than pre therapy).

Enclosed is a revised version of our manuscript “A majority of HIV persistence during antiretroviral therapy is due to infected cell proliferation”. We addressed each of the reviewers’ points in the letter below and made relevant changes in the manuscript.

We are deeply grateful for the high quality of reviews. It is obvious that each reviewer read the manuscript extremely carefully. While we slightly disagree a few of the critiques, the majority are accurate and important. We truly appreciate the feedback which will substantially improve the reproducibility, quality and clarity of our paper.

Our responses are indented below in bold:

Reviewer #1 (Remarks to the Author):

* General remarks
To distinguish between rivaling hypotheses (replication vs proliferation) for the nature of the HIV-1 reservoir under ART, Reeves et al analyze with computational methods data from other, experimental studies (Wagner et al, Maldarelli et al, etc.). These experimental studies lend support to proliferation of infected cells as major reservoir of HIV-1 diversity.

First, Reeves et al characterize the diversity of HIV-1 in the patients from the studies mentioned above using mostly standard ecological methods (Chao index, rarefaction, rank abundance curves, power-law fits). The use of ecological methods to characterize intra-individual diversity of HIV-1 is legitimate and not new (e.g. Yin et al 2012 Retrovirology 9:108, Gregori et al 2014 Bioinformatics 30:1104). The authors find that the population is dominated by clonal HIV-1 sequences, probably resulting from proliferation of infected cells. This finding is consistent with the conclusions of the experimental articles mentioned above from which the authors have taken their input data.

Secondly, to explain the discordance between apparent evolution and dominantly clonal population, Reeves et al develop a system of ordinary differential equations to model the intra-individual dynamics of infection between several compartments (actively replicating infected cells, proliferating infected cells, etc.). The takeaway of this part is that under ART the contribution of active replication and therefore evolution to observed diversity should be small if not negligible, and that apparent evolution "represents a fossil record of prior replication events".

* Main concerns

I have the following main concerns:

1. The authors state early on (line 75) the importance of studying viral evolution to distinguish between competing models (ongoing replication vs proliferation) for the HIV-1 reservoir. I agree with this statement: to test the models we have to study viral evolution, which encompasses sequence *and* population dynamics over time.

- **We agree and designed our modeling approach to be the simplest possible to represent evolution and population dynamics. By sub-setting into replication-derived populations and proliferation-derived populations, we track the fraction of cells that are likely to have clock-like evolution (those replicating) and those that will not evolve (those proliferating). We accomplish this without explicitly simulating a phylogeny, which we think provides a novel contribution and minimizes complexity.**

For instance, Lorenzo-Redondo et al 2016 Nature 530:51 (reference 4 of Reeves et al) show that observed HIV-1 sequences can be most easily explained by including ongoing evolution in the model. The clear molecular clock signal reported by reference 4 is more difficult to explain by "fossil" sequences.

- **We agree with the sentiment that the molecular clock signal in the Lorenzo-Redondo et al. (L-R) data may indeed capture ongoing HIV replication and evolution. However, we demonstrate in our paper that these observations are also consistent with distant replication events which are now embedded into a fossil record. Like all mathematical models, phylodynamic model output is inherently linked to the model's biologic assumptions. In particular, L-R et al. do not consider long-lived cells in their model, which results in a different interpretation of the evolutionary signal. Kearney et al., Brodin et al. and Rosenbloom et al. (referenced in our manuscript) argue convincingly that this assumption can bias results towards a spurious molecular clock signal. L-R et al. also do not include cellular proliferation in their model assumptions despite the fact that this is clearly a frequent event amongst reservoir cells. By considering both competing hypotheses in our ODE model, we are able to reconcile observed evolution (under certain parameter conditions) early on ART with lack of evolution after years of ART as has been observed by many studies. Our model therefore complements these papers both in model type and assumptions.**

Estimating diversity, important as it is, covers only one aspect of the system, so that, in the end, the first part of the manuscript is not more conclusive than the articles from which the authors have acquired their experimental data.

- **There is a large difference that distinguishes the present work from the original studies that we reference. In those works, though clones are discovered, the authors report that a majority of observed HIV sequences are unique singleton sequences. Here we show that while those sequences were only observed once, they are extremely likely to be members of clones. We predict that the reservoir consists of a small number of massive clones, and a massive number of smaller clones. The key to this finding lies in modeling diversity while also including population size.**
- **We thank the reviewer for this comment because we now make greater effort to emphasize the similarities and differences among ours and previous papers in the new manuscript.**

2. I am not sure how trustworthy the results in the first part (diversity estimation) are. There are a number of errors in the formulas and open questions that draw into doubt the validity of the computational procedures, for instance:
a. line 692: I am pretty sure that the binomial coefficient of x over y cannot be reasonably approximated by x^y (except for the trivial case of y=1); if the authors have used this approximation, their results are flawed

- **We apologize wholeheartedly for the errors in this equation and thank the reviewer for his/her close reading. Thankfully, the results remain unchanged because the correct approximation (as given by reviewer 3 below and now derived completely in the supplement) was used in the full calculation from which this typo factorial cancels out.**

b. line 711: I am also quite sure that the formula for sigma^2 is wrong; there should be no R^obs

- **Thank you again, you are correct and the typo is changed. The formula was computed correctly originally despite the typo in the manuscript.**

c. line 703ff: Use of Chao1 estimate: have the authors made sure that the conditions for application of Eq (3) are fulfilled (esp. homogeneity, see e.g. Chiu et al 2014 Biometrics 70:671)? Otherwise they

systematically underestimate species richness. For instance the large variance in clone size could speak against homogeneity (lines 616ff).

- **We agree with the reviewer that Chao estimator is likely to underestimate species richness based on lack of homogeneity in the data. In fact, as stated in the prior version, the Chao estimator is only intended to provide a lower plausible bound for richness in our analysis. We do not feel that tool is adequate to estimate true sequence richness in each infected person. We emphasize this point more clearly in the reviewed version.**

The authors should carefully check formulas for applicability and correctness. They should either derive formulas or cite the corresponding literature.

- **We have now included a supplementary methodology file to contain extended derivations. This was a worthwhile suggestion that strengthens the paper, and we thank the reviewer again for this and the previous comments.**

* Minor concerns

1. Lines 154-161: The authors should make sure and show that the respective experimental protocols of Wagner et al and Maldarelli et al allow that their data can be directly compared (as the authors do in Fig 2A).

- **The methods used by Wagner et al and Maldarelli et al are different. The ISLA method used by Wagner et al is lower throughput than the next generation shotgun sequencing method used by Maldarelli et al. The absolute number of viruses identified by each group differs significantly. However, in Fig. 2A we compare the percentage of singletons, which is not based on the absolute number of viral sequences identified and should be comparable across studies. Indeed, the range in percentage of singletons was similar between the two studies.**

2. Fig 2, line 208: "The proportion of observed singleton HIV DNA sequences generally decreases with time on ART" (Fig 2A)
a. Fig 2A: Binomial 95% confidence interval error bars should be shown on the points to demonstrate that suspected weak trend is likely real.
b. Fig 2A: With 5 out of 6 individuals having (weakly) decreasing singleton fractions the 95% confidence interval for decreasing singleton fractions is [0.359,0.996] based on a binomial estimate, i.e. the probability for decrease of 0.5 still is comfortably included in the 95% confidence interval.

- **This is a really good suggestion and provoked a lot of discussion internally. Our analysis demonstrates that under sampling leads to very misleading conclusions regarding the true clonal distribution of the HIV reservoir, unless our methods are not applied to the data. We also show that that the tail of the rank order distribution cannot be reliably predicted: while our conclusion that the majority of the reservoir consists of clones remains robust, we are less confident making precise estimates of the percent true singletons in the reservoir. We therefore decided that showing time trends is tangential to our main point and may cause confusion. We removed all text relating to time trends in observed data. We emphasize that our results from the model fitting should not be interpreted as an error model for these singleton fractions. The major point is that most observed singletons are likely to be clonal, and that estimating this proportion of a limited subset of the actual data will lead to massive overestimation of the percent singleton.**

c. Fig 2E and 2F and description: "In both data sets, the morphology of observed rank abundance $a(r)$ was similar across samples (Fig 2E, F), suggesting a generic structure to clonal distribution of reservoir

cells." How do the authors judge the "morphology"? Most rank abundance curves are hollow curves and thus trivially have a similar morphology. It is well possible that different cases (e.g. infected individuals but not under ART) also have "similar morphology" -- the authors should demonstrate that this is not the case, otherwise their argument of "similar morphology" is none.

- **This is a fair point. We have removed this language, and state that we simply saw a log-log linear relationship and fit the obvious simple model.**
- **Your suggestion to compare with data from participants who are not being treated with ART is really interesting. We are exploring this in ongoing work. However, this involves utilization of a completely different model and is beyond the scope of our current paper.**

3. Fig 3 and underlying formulas and descriptions
a. By "A bias-corrected estimator (avoiding division by zero)" the authors seem to imply that the purpose is to avoid division by zero. However, the modification of the Chao1 formula reduces the systematic deviation (=bias) between the richness estimate and the true richness (see Chiu et al 2014 Biometrics 70:671 and references therein). The avoidance of division by zero is a side-effect that could be achieved by other variants of Chao1, e.g. R^obs + f1^2/(2*f2+1), without changing the bias perceptibly.

- **Thank you for pointing out this semantic mistake. We have rewritten to clarify that avoiding division by zero is unrelated to the bias-corrected Chao.**

b. Line 239: What is the lower bound estimate for *observed* sequence richness?

- **Apologies, this is a typo and is corrected. We are estimating the true richness from observed data.**

4. Lines 815-817: In their system of differential equations the authors allow changes between I2 and I3 only through activation (via I1). On the other hand they state (line 405) "I2 may represent CD4+ T cells with a prolonged pre-integration phase, but their precise biology does not affect model outcomes." So, why can there be no direct link between I2 and I3? What would be the effect of such a link on the dynamics?

- **Because I2 is not a focus of our paper, but is necessary to recapitulate viral dynamics, we needed to make certain assumptions regarding I2 generation and disappearance. Moreover, it is possible to fit a tri-phasic decay including that transition because we do not have clear data quantifying the proliferation or activation rate of that compartment. That is, if we allow the transition, other rates can compensate and give the same overall kinetics of replication vs proliferation, which really is comparing I1 and I3.**

5. 75/76: Better: Due to the high error rate in HIV reverse transcription ...

- **We agree and changed text here**

6. 77-79: Inaccurate statement on mutations that should be corrected: Both the synonymous and the non-synonymous mutations are generated by error-prone replication. Some of the non-synonymous mutations are then selected by host immunity.

- **Thank you. Yours is a far better statement and we have changed this language accordingly.**

7. 234: It is unclear why authors cite reference 41.

- **We have identified a more appropriate citation.**

8. Last tick mark in Fig 2B is 2, should probably be 25.

- **Thank you. We changed the figure to avoid having this last digit cut off.**

9. Occasionally unusual wording that does not contribute to clarity, e.g.
a. 404: "we classify rapid death"
b. 668: "we developed the rank abundance curve" -> computed
c. 679: "we developed rarefaction curves" -> computed
d. 688/689/692: the "combinatorial factors" are generally known as *binomial coefficients*
e. 811: "virus is proportional" -> viral load
f. 828: "newly generated cells due to replication"

- **Thank you. These are all valid suggestions for increasing the clarity of the writing.**

10. 668: The rank abundance curve is not "r(a)", but a(r).
11. 686: missing reference or derivation
12. 688: ambiguous formula; should probably read x!/y!/(x-y)!
13. 692: missing reference or derivation
14. 695: missing reference or derivation
15. 606: no comma

- **Thank you. Each of these errors is amended in the revised version. Derivations are included in the Supporting information.**

Reviewer #2 (Remarks to the Author):

Reeves et al. analyzed data from several papers (Maldarelli, et al, Wagner, et al., and Hosmane, et al.) to determine the likelihood that the HIV-1 reservoir is maintained by ongoing viral replication in addition to clonal proliferation. They use the estimated size of the clones and the number of "singletons" from the published integration sites data to calculate the probability that any of the infected cells persisting on ART are not clonal. They conclude that that virtually all infected cells are from proliferating populations of cells. They also developed a model that allows for viral replication in ART sanctuary sites and demonstrate that, despite this, cellular proliferation will comprise the reservoir within a few months after ART initiation. This detailed analysis of published data will be an important contribution to our understanding of the HIV reservoir. I have just a few comments that I believe will improve the manuscript.

- **We really appreciate the positive feedback**

1) The exact nature of the long tail of HIV singletons is unknown and unknowable with current technology. They could say that their modeling is consistent with “all clones” but they should not say that they show that there are only clones. This would soften the conclusions which I think more appropriately fits the data.

- **We agree and apologize that our original language was too strong. Our modeling clearly shows that the tail of the distribution is unknowable with current technology and sampling limitations. Please see our response to reviewers 1 and 3 for more detail.**

2) It is not clear what is meant by the sentence in lines 146-147, "Yet, equivalent replication competent sequences are also found and at comparable frequencies." It sounds as if they are saying that intact proviruses are found in comparable frequencies as defective proviruses which is not the case. This sentence should be reworded.

- **We agree that this is poorly worded and have rewritten. Our point was that clonality is also found in replication competent sequences, and moreover, that the amount of clonality is reasonably analogous to the HIV DNA data.**

3) All of the integration sites data available in the RID were obtained from blood. It is possible that the size and frequencies of clones in tissues is different. This limitation of the analysis should be addressed.

- **This is a critical limitation which we have mentioned but now more directly address in the discussion. The entire reason for the second half of the paper that includes a possible sanctuary is to be modest regarding our conclusions of persistence based on a predominance of not sampling lymph tissue. While we believe that existing literature supports the idea that blood sequences are generally representative of sequences in lymph nodes and GALT, the possibility of sanctuaries that never are reflected in blood is extremely hard to rule out. Indeed, if blood sequences are 100% uncorrelated with tissue sequences, our conclusions may not be accurate. This is a struggle for the entire field unfortunately.**

4) Although it may not affect the outcome model, the statement that a minority of proviruses are latently infected in pre-ART is not correct for patients who initiate ART in chronic infection. Latently infected cells accumulate on ART and are the majority after years of infection even prior to starting treatment. It is more likely that the cells that die in the first months include those that are readily reactivated. Those that were not latent pre-ART likely die within days of initiating ART.

- **Thank you for this interesting point. In order to fit to the sequential viral load data in Figure 6, we must make assumptions about the size of the three infected cell compartments as well the reactivation rates of I2 and I3 to I1. In order to fit the data, the size of I2 and I3 and their reactivation rates are inversely correlated. Because there are no experimentally verified measures of I1, I2 or I3 prior to ART, or of the reactivation rates, our assumptions of the relative size of the I1, I2 and I3 compartments are estimates only. We highlight this in the revised version.**
- **To assess whether this uncertainty impacted the scientific conclusions of our model, we simulated the model with differing assumptions regarding the volume of I3 from 10^5-10^7 cells (adjusting the reactivation rate accordingly) and identified that proliferation sustains the reservoir under each of these parameter regimes.**

5) Current integration sites data is still limited and, as a result, some of the conclusions are stated a bit strongly and should be softened. However, it should be more strongly stated that these results are in direct opposition to the Lorenzo-Redondo, et al. conclusions that the reservoir is sustained by ongoing cycles of viral replication.

- **Thank you. We agree wholeheartedly and emphasize in the discussion that integration site data, while robust, is limited to a handful of participants in only a few studies.**

Reviewer #3 (Remarks to the Author):

Overall:

HIV persists despite long-term antiretroviral therapy, and the mechanism for this persistence continues to be debated. The major proposed mechanisms are a) ongoing replication of virus, despite drugs, in a “sanctuary” site in the body, b) long lifespans of latently infected memory CD4+ T cells, c) proliferation - without virus production and cell death - of these latently infected cells. In this paper the authors use two distinct methods to suggest that (c) is the dominant mechanism. The first part uses an analysis of

the clonal composition of cells in the latent reservoir - based on uniquely identifying the virus infecting them and showing that many latent cells are descendants of the same infection event - to argue that latent cell proliferation must be maintaining the reservoir during ART. The second part is a mathematical model of both ongoing HIV replication in a sanctuary and HIV persistence in latent cells, and puts limits on how much ongoing replication could contribute to the observed # of latently infected cells.

Altogether, I think both of the authors arguments are strong and that their unique approach is an important contribution to this debate, which is critical to deciding the future of HIV therapeutics. The authors inform their models with data wherever possible and consider a range of possible scenarios and parameter values to evaluate the robustness of their conclusions. Overall their conclusions agree with those of many other papers (which they reference), but they have arrived at them by novel means, leading further credence to them.

- **We appreciate the positive feedback.**

I have a few suggestions for how the authors could improve this work. As I outline below, there are multiple areas where explanations of the models and results are confusing and could be improved. More importantly, I think the authors need to do a better job of providing a coherent transition between the two very separate analyses in their paper (statistical analysis of clonality of reservoir from genetic data and mathematical model of ongoing replication vs latent cell dynamics). There isn’t really any connection between these two halves of the paper, except that they are both related to the mechanism of HIV persistence during ART.

- **Thank you for this insight. We acknowledge that the transition between the two sections needs improvement. In the revised version we more clearly highlight that we are first demonstrating how data indicate that proliferation sustains the reservoir after 1 year of ART. We then use a mechanistic mathematical model to explain why this observation is not at all incongruent with observations of evolution earlier during ART. The mathematical model specifically allows the possibility of two generative mechanisms of infected cells including cellular proliferation and HIV replication. In combination, our analysis highlights that reservoir studies during the first year of ART need to include the assumption of long-lived, proliferating cells and as such, represent a fossil record of historic evolutionary dynamics.**

In addition, they authors could do a better job - at minimum in the Discussion - of acknowledging the major assumptions and hence limitations of their work. The conclusion of the first half of the paper is almost entirely based on assuming that the clone size distributions follow a particular model and extrapolating this model out to the entire reservoir composition. Of course there are many reasons why this model may not be correct.

- **This is an excellent point. Biologically speaking, sequences derived from blood samples may not accurately represent those derived from lymphatic tissues. Mathematically speaking, the power law may not extend to minority species not observed in blood, but that are nevertheless critical for maintenance of the reservoir. These limitations are insurmountable without much deeper sampling of viruses from multiple tissue sites and we acknowledge this more forcefully in the revision. We again mention that autopsy studies represent the only plausible method to completely verify the predictions of our approach. We do feel that our method is robust for drawing conclusions about majority sequences in blood.**

The conclusion of the second half is based on the assumption that target cell availability in a proposed sanctuary actually declines during ART, an idea which has so far not been including in any other models of HIV dynamics and is hard to directly provide experimental evidence for, since no one really knows

which subset of CD4+ T cells is actually susceptible to HIV (only a very small fraction of cells are ever infected even during peak viremia).

- **This is again a very fine point. The concept that HIV increases its own replication in the absence of ART by activating local CD4+ T cells (and that ART eliminates this positive feedback loop) is commonly discussed and makes logical sense but is experimentally unproven. To accommodate this uncertainty, we feel that the fairest approach is to simulate the model with and without this assumption. As now stated in the current version of the model, even when the model is simulated without the assumption of a slowly contracting population of target cells, we estimate that after one year of ART (assuming a large drug sanctuary), a majority of new infected cells would be generated via proliferation. Of note, in our prior simulations the rate of target cell depletion was very, very low and reflected the overall decrease in the abundance of these activated CD4+ T cells on ART.**

I think that the authors can easily make the suggested changes to the paper and that it will be a very appreciated contribution to this important debate in the HIV field.

- **Thank you again.**

Methods:

~668: Should explain that “abundance” in rank-abundance curves refers specifically to the relative proportion of the population composed of a particular clone
~686: You haven’t yet explained what capital R stands for

- **Thank you for noticing these details, we have rewritten substantially. In particular we removed the proportional abundance to avoid confusion with absolute abundance.**

~670: Throughout the paper, but especially here, the way in which the distribution of clone sizes is manipulated to generate these derivative distributions such as "rank abundance" and "rarefaction curves” is quite confusing and the relevant terms are not defined clearly. For example, what is Robs? I thought it was the number of distinct clones observed in a sample. But, in line 684, you describe in words a quantity that seems exactly to be R_obs, and then give a formula for it, but this formula involves R_obs! So what is the thing you are defining in this line vs what is R_obs? Similar point for Equation 2. And what is the difference between k and N? Which is the sample size and what is the total population size? Both are referred to as sample sizes in the text. And what is Nobs and how does it differ from N? Is this formula referring to resampling the sample or sampling the population? Something cannot be an equation without having an equal sign with something on both sides. So what quantity is being defined by each of these formulas? I think the left hand side should say "R_obs(k) =" and the right hand side should be your formula but “R_obs” should be replaced by “R_obs(N_obs)”.

- **Upon rereading, we agree with you and apologize for the confusion. We have rewritten this section substantially. Because these concepts are quite complex, we have included a new supplementary file that puts extra focus on precise definitions and derivations.**

I think it is worth pointing out that a “rank abundance curve" is actually just a probability distribution/histogram - all the quantities add up to 1. I think “rank abundance distribution” would be a better name than “rank abundance curve” for this reason.

- **While rank abundance curves can be probability distributions, we now only display them in terms of real population sizes. This helps to emphasize the point that current experiments are under sampling the reservoir leading to observed singletons which are likely to actually be**

members of larger clones. Referencing the comment above, we have included extra discussion about our definitions in a supplementary file.
- **Also, we find the reviewer correct that making the link with a histogram helps intuition and have made this point clear in the methods.**

~692: This approximation for the binomial coefficients is not correct.
See https://math.stackexchange.com/questions/1447296/stirlings-approximation-for-binomial-coefficient for correct formula.
- **The reviewer is of course correct. Apologies for the typo, it is changed.**

Re Chao estimator: It would be nice to have a bit of an explanation for what is the theoretical basis for this estimator, and why you can claim it is "validated".
- **In the methods, we have added text and further references discussing the past uses.**
- **We also add text to address Reviewer 1's concern about the Chao as a minimum estimator.**
- **Finally, we include a derivation of the estimator in the supplementary file.**

~736: The method described for fitting the proposed log distribution to the observed rank abundance distribution is not very statistically rigorous. Sum of squared errors approaches are not appropriate for CDF as they are more heavily weighted by values far from 0 and 1, and your current method is limited by the increments of parameter values you choose and the number of sample draws you simulate. Also, it does not properly take into account the sampling process. A more formally correct way would be to write down a likelihood function for a given observed set of data under give model parameters, and then either use formal optimization techniques to maximize the likelihood, or, use a Bayesian approach and sample from the posterior using MCMC to generate an estimate - with credible intervals - for each parameter value.
- **This is a fair point and one which we have had substantial internal debate. A major reason we developed this fitting procedure is that we have attempted to account for the sampling process. The process of sampling that leads to rank abundance distributions does not lend itself well to analytical likelihood calculations. While we attempted to explain this in the supplementary figure on the methodology originally, we now walk the reader through the process with visuals in the supplementary file.**
- **We agree that the residuals of the fitting are not symmetric over the interval 0-1. We note that another approach to fitting cdfs is the Kolmogorov-Smirnov statistic. This statistic has its own challenges (sub groups of data can give different results), but we note that this statistic provides very similar results to our approach. It uses the maximum deviation between cdfs to score fit, and because the rms errors are not symmetric, they are determined mostly by the maximum deviation.**
- **Overall, we believe our demonstration of the model working accurately on simulated data (now discussed in more detail in the supplementary information) is sufficient evidence that our methods are appropriate for our admittedly coarse fitting.**

Line 789 - I think the analysis in Luo et al (http://rsif.royalsocietypublishing.org/content/10/84/20130186) is much more trustworthy source of estimates for the size of a compartment for residual replication, given that Lorenzo-Redondo et al is based on faulty estimates of reservoir evolution when in fact they were not measuring the long-lived reservoir at all.

- **We agree with this assessment that the Lorenzo Redondo paper is likely to be measuring a fossil record of prior replication events. The Luo paper measures 2-LTR dynamics after treatment intensification with an integrase inhibitor and concludes the presence of a drug sanctuary producing 10-310 million new infected cells per day in the 7 of 45 participants who had a signal consistent with cryptic viremia. Therefore, for most ART treated person, our assumption that a sanctuary would need to be small enough to avoid detection with routine sampling remains valid. From another point of view, Luo et al conclude that the degree of residual infection predicted by their model would be enough to generate resistant mutants. Therefore, it is possible that the 7 participants in their study were developing treatment failure. We now reference the Luo paper, but because of the complexity of their model and experimental nuances, we keep the direct comparison to L-R et al. In fact, we believe using unrealistically large estimates for a sanctuary only strengthens our argument.**

Equation 6: This model is extremely poorly explained here. It is described very clearly in the main text, but then if someone is just reading the methods, there is no explanation. It would be good to either refer readers back to the main text, or repeat some of it here. More generally, the "Results" and the "Methods" section each contain a random subsample of the methods - might be more coherent to keep them in one place.

- **We agree, and have simplified the writing in the results and moved much of the detail to the methods. Hopefully the flow is better now and the information is organized more appropriately between sections. We also include an additional section on this model in the supplementary file to completely describe the model and open source code that is freely available to reproduce all simulations the manuscript.**

~821: These is something grammatically wrong with the first (non-bolded) sentence that makes it nonsensical. What does this formula mean? What are the numerator and denominator? Since you haven't mentioned anything about proliferation yet it is totally unclear.

- **Thank you for pointing this out. We now write substantially more about these formulae.**

Eq 9 - 11 : Do not understand what is going on here.

- **We now provide more detail regarding derivation of equation 11.**

~853: Explain what these parameters are and what these ranges mean (where did they come from, what units are they in, etc).

- **Thank you. We now include details to facilitate interpretation of the parameters.**

Introduction:

~75-78. There are a few misleading statements about evolution here. Firstly the sentence that starts with "Due to .." does not really make sense. The factors mentioned at the beginning of the sentence are not why evolution implies replication. Evolution implies replication because we assume that the only way in which the genetic composition of a population can change is by mutation and selection, and we assume both require replication and thus turnover of a population. Note that this is not actually true though … there are other reasons why genetic composition could change … e.g. migration, differential death rates, etc. In regards to the next sentence, the high mutation rate of HIV RT is what is responsible for generating a huge amount of diversity on which selection can act. Immunological "pressure" is what actually facilitates selection. The large population size means that selection can be efficient, when

compared to drift. For both synonymous and non-synonymous mutations, the mutations themselves are generated by error prone replication. The latter can be subject to selection.

- **We agree and have reqritten this section of the introduction to capture the effects viral diversification and positive selection pressure.**

~84: I think that the reference to 18 here is incorrect. I don't think Rosenbloom et al commented on undersampling .. their point was about differential decay of infected cells (exactly as you've included in your later model) masquerading as evolution during early ART initiation.

- **We agree and have separated this sentence into 2 sentences to discriminate the points made in the Kearney article (PCR resampling and limited dataset sample size) versus the Rosenbloom article (differential decay rates of infected cell subsets).**

Results:

The heading of the section starting at 197, and the title for the caption of Figure 3, seem to be overstatements. You do not actually have proof of either of these statements. They are simply predictions of your model.

- **While these are model predictions, they are highly likely to be true based on the data alone. As an extreme example, the presence of a single doubleton would make the statement that "The total number of HIV DNA sequences in the HIV reservoir exceeds the total number of unique sequences" true. Nevertheless, we change several section heads and figure legends by adding the prefix that "Modeling suggests…"**

The Chao estimator should probably be described briefly here - what assumptions does it make and what theory is it based on? How does it make a prediction for the total species richness?

- **As described above, we now include more details on the Chao estimator including its derivation in the supplementary information.**

Line 269 - 278 : This fitting method is already described in the Methods and doesn't have to be mentioned in such detail here. Just say that you fit the observed distribution to a log-linear function to determine the best-fit parameters.

- **We have changed this section accordingly.**

Figure 4: The difference between panels C/D vs E/F is not clear from the caption. Same for Figure 5.

- **We added sufficient detail to the caption in the revised version.**

~404: It's not clear to me why you decided to include in your model the complicating factor that target cells decline during ART. It seems like this is biasing towards your hypothesis that ongoing replication is not important. Why bother including this? And why bother including the multiple subtypes of latently infected cells?

- **It is critical to include the latently infected subtypes because there is strong empirical data that these cells proliferate on average at different rates. Inclusion of slowly proliferating subsets biases against proliferation as the dominant birth process and makes the interesting point that increasing the numbers of long lived latently infected cells will extend the impact of the fossil record.**
- **Target cell decline seems like a reasonable assumption to us based on the decline of activated CD4+ T cells during ART. However, as described above, there is no experimentally verification**

that a decline in the overall decline in activated CD4+ T cells during ART is sufficient to limit target cell availability. Therefore, in the revision, we simulate the model with and without this assumption.

- **Importantly, we draw the conclusion that we cannot exclude a small drug sanctuary which produces new sequences (infected cells) but does not interact with other compartments. While proliferation remains the primary generative force for new infected cells, we cannot eliminate the possibility that better ART delivery will be needed to eliminate a tiny minority of persistent infected cells.**

Figure 7: I don't understand what "net replication percentage" is

- **This denotes the birth process of all observed cells (not new infected cells). We have substantially rewritten to make these distinctions clear.**

In line 458 you mention a sanctuary size of 0.1% (e.g. 1 in 10^3), but then in Figure 7 you only show much smaller sanctuary sizes - 1 in 10^5 and 1 in 10^6. Why? Wouldn't you conclusions about relative % of cells from new replication change if you used larger sanctuary sizes? Isn't the sanctuary size constrained by the total amount of residual viremia observed during ART? (around 1 copy/mL, which is ~10^4 lower than pre therapy).

- **We agree and have eliminated this discrepancy. As discussed above, we constrain the sanctuary to according to observed viremia (with acknowledgement of the 2-LTR data from Luo et al) which is about 1/10,000 – 1/100,000 or 0.01-0.001%.**

Reviewers' comments:

Reviewer #1 (Remarks to the Author):

* Major

- line 548: "Estimates of the size of the HIV reservoir (both replication competent and total) were gathered from the published literature.[33]".
- In Ref 33 (Ho et al 2013 Cell 155:540) I could not find the values for L used by the authors. Ref 33 shows that the estimated reservoir sizes vary "dramatically" (i.e. by orders of magnitude) between patients.
- The authors should make clear where L=10^9 comes from that they use throughout their text and what the consequences of the dramatic variations of L between patients reported by Ref 33 are on their estimations.

- line 541: "so that 2 standard deviations (~95% confidence intervals) are given by 2sigma" and line 542: "These are small in general relative to R^Chao1 so we did not include these values in our plots".
- Underlying the statement in line 541 is usually the assumption of normally distributed richness values. In this case, the width of the 95% confidence interval is 4sigma, not 2sigma.
- If I compute R^Chao1 from Eq 3 and sigma = sqrt(sigma^2) from Eq 4, I cannot confirm the statement that sigma or the width of the 95% confidence interval is small in comparison to R^Chao1. For instance, for individual S06 I obtain R^Chao1 = 118 and sigma approx. 220, and 4sigma approx. 880 for the width of the 95% confidence interval. Thus the 95% CI is by no means negligible in comparison to R^Chao1. The fact that the error bars cross into negative abundance values shows that the approximations used here are invalid. I tried this also for S03 and came to the same conclusion that contradicts the statement of the authors that "these are small in general relative to R^Chao1".
- In Eq S13 the authors give instead an asymmetric 95% confidence interval for R^Chao1 that differs from the one given in the Methods (Eq 4 etc.). If we apply Eq S13 we still obtain values for confidence interval widths that are larger than R^Chao1 and thus not negligible, again contradicting the statement of the authors.
- The authors should use a consistent expression for the confidence interval and report confidence intervals.

* Minor
- l.50: distance between DNA sequences should usually not be measured by the Hamming distance because of indels.
- l.77: "same ... locus by chance alone": Is it clear that integration is governed by chance alone? Are there references?
- l.110: allows -> allow
- l.151: a(1) is no clone but the size of a clone
- l.276: identifies -> identify
- l.326: reformulate "relatively equivalent"
- l.429: replace "instead"

- l.470/471: limited, limited
- l.576: reformulate "all possible richness"

Reviewer #3 (Remarks to the Author):

Review 2:

Overall I think the authors have dramatically improved the clarify of the paper with this revision. They have responded to all my comments and have also fairly addressed the comments of all the other reviewers. I agree with the authors, in their rebuttal to Reviewer 1, that Lorenzo-Redondo et al provides no real evidence of ongoing replication (vs other mechanisms like the fossil record). As with the first draft, I think this is important work which will really help the HIV field understand mechanisms of persistence during ART. I recommend it for publication.

I have one minor comment and one more substantive comment:

Minor:

Line 434: I don’t really understand this sentence "While at this time, sequences only originate from and via reactivation, ongoing evolution might be observed due to the relatively equivalent cell population sizes.”. Overall this paragraph is very unclear. The contents do not seem to provide any support for the bolded first sentence

More substantive:

I don’t fully agree with the analysis related to the transition from replication to proliferation as the dominant source of new cells. The authors insist on mainly analyzing their viral dynamics model with decay of activated cells, which is very non-standard. Even if there is some evidence for decrease in activated CD4 when ART is started, it is extremely unlikely that this % just continually decreases towards zero. Much more probable is that it decreases a bit then levels off. Under the decaying target cell model model, the authors claim that their conclusion that "after ~<1 year on ART most infected cells are from proliferation, not replication" is robust to large variations in any other parameters. Then they also claim, in Line 521, that “Our analysis included a sanctuary in which target cell availability did not decay at all. In that scenario, 5-10% of new infected cells were generated by HIV replication after a year of ART (Fig 9A), which is not consistent with lack of viral evolution observed at this timepoint.”

However, this is all very misleading. Clearly, if there is no decay in target cells in the sanctuary, then any conclusion about the fraction of infections due to replication is completely dependent on the assumption about the size of the sanctuary relative to the size of the latent reservoir. And these two sizes are just numbers the authors choose. So no model is even needed here. It is intuitively clear that if target cells in the sanctuary decay

with the high rate used here (by about 3 orders of magnitude in 1 year with the values used here, based on orange line in Figure 6C), then no matter what other parameters are used, pretty quickly the sanctuary will become unimportant. And it is also obvious that if they don’t decay, then the relative size of the sanctuary vs reservoir (and of course the proliferation rate of the reservoir), determine the relative importance of proliferation vs new infection when an approximate steady state is reached (once I1 and I2 cells are gone).

Furthermore, I disagree with this concluding determent (line 613): "If we do not allow the sanctuary to contract as a result of target cell decline, a persistent low-level sanctuary emerges that stabilizes at 6 months and generates ongoing evolution at later ART time points.” This is just not true. It is only true for the parameter values you chose for sanctuary size and reservoir size. You do not know what these values really are! All you know is that the sum of these values must equal total HIV DNA, and that the sum of RNA released from newly infected cells in sanctuary plus RNA from cells reactivated from latency must equal residual viral load (which is generally undetectable). Your estimate for reservoir size comes from assuming ALL HIV DNA comes from the reservoir (so then the fact that it would dominate is a foregone conclusion), and your estimate for size of sanctuary is from a paper who’s analysis was totally flawed (Lorenzo-Redondo et al) for reasons the authors highlight in this very paper!

Despite this, I think the authors should decide how to present this issue and I am not going to hold back publication based on this. However, I think it would be much more honest to investigate and present the robustness of their conclusions (proliferation vs new infection) to parameters that are actually expected to influence the relative importance of these two processes.

Enclosed is the second revised version of our manuscript "A majority of HIV persistence during antiretroviral therapy is due to infected cell proliferation". We addressed each of the reviewers' points in the letter below and made relevant changes in the manuscript.

We also noticed a small error in compiling the integration sites, and reran all analyses in the first section, which resulted in some small changes to numbers. In the paragraph at line 172, 1-19%, mean 10% becomes 1-16% (mean: 7%), from 1-83 mean 12 becomes 1-150 (mean: 15), 3-62 sequences (mean: 11), accounting for 3-32% (mean= 9%) becomes 2-62 sequences (mean: 11), accounting for 3-26% (mean= 9%).

Again, we are grateful for the high quality of reviews and appreciate the feedback, which has substantially improved the reproducibility, quality and clarity of our paper.

Our responses are indented below in bold:

Reviewer #1 (Remarks to the Author):

* Major

- line 548: "Estimates of the size of the HIV reservoir (both replication competent and total) were gathered from the published literature.[33]". In Ref 33 (Ho et al 2013 Cell 155:540) I could not find the values for L used by the authors. Ref 33 shows that the estimated reservoir sizes vary "dramatically" (i.e. by orders of magnitude) between patients. The authors should make clear where L=10^9 comes from that they use throughout their text and what the consequences of the dramatic variations of L between patients reported by Ref 33 are on their estimations.

It is true that the measurements from Ho et al. vary considerably. We chose the median value from that paper for DNA, which agrees fairly well with the ranges and median from Besson et al. (ref 47). While the replication competent reservoir is often cited at 1e6 cells, we chose a higher value (inclusive of noninducible provirus in ref 33) to bias against our conclusions. Most importantly, as we note, beyond roughly R>10^6, the value of R was not identifiable. Choosing a different value for L thus changes R proportionally. Because we show that the richness divided by the total population size is relatively small (R/L~1/100) using different values of L does not affect those conclusions. We have now included language to this effect (lines 242 and 303).

- line 541: "so that 2 standard deviations (~95% confidence intervals) are given by 2sigma" and line 542: "These are small in general relative to R^Chao1 so we did not include these values in our plots". Underlying the statement in line 541 is usually the assumption of normally distributed richness values. In this case, the width of the 95% confidence interval is 4sigma, not 2sigma. If I compute R^Chao1 from Eq 3 and sigma = sqrt(sigma^2) from Eq 4, I cannot confirm the statement that sigma or the width of the 95% confidence interval is small in comparison to R^Chao1. For instance, for individual S06 I obtain R^Chao1 = 118 and sigma approx. 220, and 4sigma approx. 880 for the width of the 95% confidence interval. Thus the 95% CI is by no means negligible in comparison to R^Chao1. The fact that the error bars cross into negative abundance values shows that the approximations used here are invalid. I tried this also for S03 and came to the same conclusion that contradicts the statement of the authors that "these are small in general relative to R^Chao1". In Eq S13 the authors give instead an asymmetric 95% confidence interval for R^Chao1 that differs from the one given in the Methods (Eq 4 etc.). If we apply Eq S13 we still obtain values for confidence interval widths that are larger than R^Chao1 and thus not

negligible, again contradicting the statement of the authors. The authors should use a consistent expression for the confidence interval and report confidence intervals.

Thank you for these excellent detailed points. We apologize for the imprecise language and clarify that in our response we meant that the confidence intervals appear small on the logarithmic scales on which they are plotted so that they are fairly negligible relative to other estimates. We now include the asymmetric intervals in Fig 3. The discrepancy in equations was a weak attempt to use a simpler equation in the text and expand in the supplement, however, it is much better to keep it consistent, so the full equation is described in the Supplementary information. Overall we have expanded the confidence intervals for all of our biological conclusions because we bound the richness between the Chao estimate of minimum richness and our model estimate of maximum richness based on reservoir size.

* Minor
- l.50: distance between DNA sequences should usually not be measured by the Hamming distance because of indels.

Indeed it is more complicated. We have changed the line to "often measured by continual growth in genetic distance between the consensus strain and the founder virus"

- l.77: "same ... locus by chance alone": Is it clear that integration is governed by chance alone? Are there references?

We have changed the language here to be more precise and include references about the specifics of HIV integration.

- l.110: allows -> allow
- l.151: a(1) is no clone but the size of a clone (removed on line 179)
- l.276: identifies -> identify (now 364)
- l.326: reformulate "relatively equivalent" (paragraph removed)
- l.429: replace "instead" (now 571)
- l.470/471: limited, limited (now 611)
- l.576: reformulate "all possible richness" (now 711)

Thank you for catching these mistakes or clarity issues, wording is amended to fix grammar and new line numbers are added to verify changes in the new document.

Reviewer 3
Overall I think the authors have dramatically improved the clarify of the paper with this revision. They have responded to all my comments and have also fairly addressed the comments of all the other reviewers. I agree with the authors, in their rebuttal to Reviewer 1, that Lorenzo-Redondo et al provides no real evidence of ongoing replication (vs other mechanisms like the fossil record). As with the first draft, I think this is important work which will really help the HIV field understand mechanisms of persistence during ART. I recommend it for publication.

We greatly appreciate Reviewer 3's enthusiastic feedback.

I have one minor comment and one more substantive comment:

Minor:

Line 434: I don't really understand this sentence "While at this time, sequences only originate from and via reactivation, ongoing evolution might be observed due to the relatively equivalent cell population sizes.". Overall this paragraph is very unclear. The contents do not seem to provide any support for the bolded first sentence

This paragraph has been completely rewritten based on your feedback below, see line 425 and the following paragraphs.

More substantive:

I don't fully agree with the analysis related to the transition from replication to proliferation as the dominant source of new cells. The authors insist on mainly analyzing their viral dynamics model with decay of activated cells, which is very non-standard. Even if there is some evidence for decrease in activated CD4 when ART is started, it is extremely unlikely that this % just continually decreases towards zero. Much more probable is that it decreases a bit then levels off. Under the decaying target cell model model, the authors claim that their conclusion that "after ~<1 year on ART most infected cells are from proliferation, not replication" is robust to large variations in any other parameters. Then they also claim, in Line 521, that "Our analysis included a sanctuary in which target cell availability did not decay at all. In that scenario, 5-10% of new infected cells were generated by HIV replication after a year of ART (Fig 9A), which is not consistent with lack of
viral evolution observed at this timepoint."

However, this is all very misleading. Clearly, if there is no decay in target cells in the sanctuary, then any conclusion about the fraction of infections due to replication is completely dependent on the assumption about the size of the sanctuary relative to the size of the latent reservoir. And these two sizes are just numbers the authors choose. So no model is even needed here. It is intuitively clear that if target cells in the sanctuary decay with the high rate used here (by about 3 orders of magnitude in 1 year with the values used here, based on orange line in Figure 6C), then no matter what other parameters are used, pretty quickly the sanctuary will become unimportant. And it is also obvious that if they don't decay, then the relative size of the sanctuary vs reservoir (and of course the proliferation rate of the reservoir), determine the relative importance of proliferation vs new infection when an approximate steady state is reached (once I1 and I2 cells are gone).

Furthermore, I disagree with this concluding determent (line 613): "If we do not allow the sanctuary to contract as a result of target cell decline, a persistent low-level sanctuary emerges that stabilizes at 6 months and generates ongoing evolution at later ART time points." This is just not true. It is only true for the parameter values you chose for sanctuary size and reservoir size. You do not know what these values really are! All you know is that the sum of these values must equal total HIV DNA, and that the sum of RNA released from newly infected cells in sanctuary plus RNA from cells reactivated from latency must equal residual viral load (which is generally undetectable). Your estimate for reservoir size comes from assuming ALL HIV DNA comes from the reservoir (so then the fact that it would dominate is a foregone conclusion), and your estimate for size of sanctuary is from a paper who's analysis was totally flawed (Lorenzo-Redondo et al) for reasons the authors highlight in this very paper!

This is a very fair critique and we really appreciate the reviewer's thoughtful input. We are persuaded by much of the reviewer's argument. Because a drug sanctuary has not been observed, its theoretical size and decay characteristics are inherently unknown (if a sanctuary even exists). However, as the reviewer points out, it is fair to put an upper bound on the size of a theoretical sanctuary based on the vast reduction in detectable HIV RNA during effective ART. In fact, the idea that the sanctuary might be decaying was only added originally to bias against our argument, as you will now see, if the sanctuary is small enough to not be observed during ART, it must have been an extremely small fraction of infected cells pre-ART.

We wish to include the second half of the paper for two reasons.

1) Our approach in the first half does not account for the mechanistic cellular dynamics which might underlie the observed data. Using a mechanistic model allows us to actually quantify the wash out period for the fossil record, which we believe could be useful to inform future experiments (~6mo to 1 year seems ideal). Our model also explains the transition between (alleged) observed evolution early on ART and observed clonality late on ART. There is yet no data in this regime so our model interpolates.

2) The model allows us to emphasize the discrepancy between true mechanism and observed mechanism of persistence. This point is hard to make (we find) with writing alone, but with the model, it is clear that there is an inherent lag in the observed data due to varying age structure of observed sequences (the fossil record).

In this version, we revised Figures 6 & 7 to highlight that these results are consistent whether or not a small sanctuary is assumed, and further if it were assumed to be large initially but decaying with a rate that would still lead to the observed viral decline. We only include the concept of target cell decay in the sensitivity analysis to highlight the theoretical fact that completely different biologic processes would drive trends in the observed data versus the actual ongoing processes sustaining the reservoir. We feel that this is important to include to emphasize that interpretation of phylogenetic trees, in the absence of a deep understanding of the underlying viral and cellular dynamics driving the system, can lead to misinterpretation of the data.

It is important to emphasize that neither of our approaches can rule out the persistence of a tiny drug sanctuary which contributes to a miniscule percentage of persistent HIV. However, the two approaches in combination suggest that all available observed data (including Lorenzo-Redondo et al. 2016) is entirely consistent with the fact that cellular proliferation underlies reservoir persistence.

Despite this, I think the authors should decide how to present this issue and I am not going to hold back publication based on this. However, I think it would be much more honest to investigate and present the robustness of their conclusions (proliferation vs new infection) to parameters that are actually expected to influence the relative importance of these two processes.

Thank you for this excellent discussion. We agree that making certain assumptions about target cell depletion is premature and include this only to suggest a possible mechanism that might allow for a larger sanctuary initially, highlighting the vast differences between observed data and contemporaneous processes generating infected cells. We hope that the revised version clearly narrows the scope of conclusions related to the model, and that these conclusions are valid whether a sanctuary is assumed or not.

REVIEWERS' COMMENTS:

Reviewer #1 (Remarks to the Author):

The authors should check their figures. Several figures in the PDF file, e.g. 4E and 5E, give the impression of having been partially overplotted.

Otherwise ok.

Reviewer #3 (Remarks to the Author):

The authors have addressed all my comments

REVIEWERS' COMMENTS:

Reviewer #1 (Remarks to the Author):

The authors should check their figures. Several figures in the PDF file, e.g. 4E and 5E, give the impression of having been partially overplotted.

Otherwise ok.

This is a good point, we have changed the transparency and shifted lines slightly so that overlapping lines are now visible.

Reviewer #3 (Remarks to the Author):

The authors have addressed all my comments

Thanks for all the great suggestions.
