# Supplementary material for: A majority of HIV persistence during antiretroviral therapy is due to infected cell proliferation
Source: Nat Commun. 2018 Nov 16;9:4811. doi: 10.1038/s41467-018-06843-5 (PMC6240116; doi:10.1038/s41467-018-06843-5)
Supplement: Supplementary file 1 — Peer Review File [file 41467_2018_6843_MOESM1_ESM.pdf]

Supplementary Information: A majority of HIV persistence during antiretroviral therapy is due to infected cell proliferation

DB Reeves, ER Duke, TA Wagner, SE Palmer, AM Spivak, JT Schiffer

Description

In this supplementary information we include additional derivations and model fitting explanations. All code is freely available at `https://github.com/dbrvs/reservoir_persistence`.

Contents

1 Supplementary Methods **2**
- 1.1 Rank-abundance distributions . . . 2
- 1.2 Population level rank abundance with compiled data . . . 2
- 1.3 Estimating richness non-parametrically with the Chao1 estimator . . . 2
- 1.4 Deriving the Chao1 estimator . . . 2
- 1.5 Chao1 confidence interval . . . 4
- 1.6 Derivation of Cauchy-Swartz inequality . . . 5
- 1.7 Sample rarefaction . . . 5
- 1.8 Modeling rank-abundance distributions (power law) . . . 7
- 1.9 Approximating the normalizing constant by integration . . . 7
- 1.10 Model fitting procedure, example with simulated data . . . 8
- 1.11 Other functional forms map to power-law, e.g. log-normal . . . 9

2 Supplementary Figures **11**

1 Supplementary Methods

1.1 Rank-abundance distributions

The data we use are counts of HIV sequences. A 'sequence' is defined by its integration site to the host genome in the Maldarelli et al. and Wagner et al. data [1, 2], or by its HIV sequence in the Hosmane et al. data [3]. We arrange these data so that a given sequence is given a rank r, determined by the number of identical sequences. The maximum rank defines the richness R (the number of species in an ecological context). The ranks are discrete such that $r \in [1, 2, \ldots, R]$. A group of identical sequences is referred to as a population of sequence clones. Thus the abundance of all ranked sequences is the rank-abundance $a(r)$, which also takes on discrete integer values. We also at times use a normalized rank-abundance referred to as proportional abundance $p(r)$, and additionally a cumulative proportional abundance, or cpa, $\sum_r p(r)$.

1.2 Population level rank abundance with compiled data

By compiling data from all participants in the total HIV DNA and replication competent DNA studies, we created two population level observed rank abundances. These data were used in the same fitting procedure as the separate participant data to bolster sample size and check for connection between population and individual clonality. Results are mentioned in the main text, and shown in **Supplementary Fig 3**.

1.3 Estimating richness non-parametrically with the Chao1 estimator

To develop an estimate for the minimum true richness R based on the observed data we use a non-parametric estimator based upon the observed frequencies of clonal populations of different sizes first described by Chao [4]. There are several excellent reviews of this type of estimator, see e.g. Ref. [5].

1.4 Deriving the Chao1 estimator

To derive the Chao1 estimator, we envision an experiment as a sampling process where R^{obs} distinct sequences were independently sampled without replacement from a very large population of HIV sequences. The probability of observing the r-th sequence k times after N samples is given by a binomial sampling process

$$P(a(r) = k) = \binom{N}{k} p_r^k (1 - p_r)^{N-k} \tag{S1}$$

where p_r is the true proportional abundance of the r-th sequence clonal population. We do not specify these probabilities here. $\binom{N}{k} = \frac{N!}{k!(N-k)!}$ is a binomial coefficient.

We call unique sequences (clonal population size 1) singletons. The number of singletons is N_1. Sequences observed twice (clonal population size 2) are doubletons, and so forth. The number of doubletons is N_2. Onward, N_k represents the number of k-tons or the number of clonal populations with k members. From our experiment, the number of sequences observed k times can be computed

$$N_k = \sum_{k=1}^{R^{obs}} \binom{N}{k} p_r^k (1-p_r)^{N-k} \tag{S2}$$

Following Ref. [6], our derivation of the Chao1 estimator uses a Cauchy-Schwarz relationship between N_0 and N_2. We begin by defining two suggestive vectors:

$$u_i = \sqrt{(1-p_i)^n}, \quad v_i = \sqrt{p_i^2(1-p_i)^{n-2}} \tag{S3}$$

In the N dimensional Euclidean space $\mathbb{R}^N$, the Cauchy-Shwarz inequality relates the magnitudes or Euclidean norms $||x|| = \sqrt{\sum_{i=1}^N x_i^2}$ of these vectors as

$$\left[\sum_{i=1}^N u_i v_i\right]^2 \leq \left[\sum_{i=1}^N u_i^2\right] \left[\sum_{i=1}^N v_i^2\right]. \tag{S4}$$

Inserting our vectors and collecting and simplifying exponents leads to

$$\left[\sum_{i=1}^N p_i(1-p_i)^{N-1}\right]^2 \leq \left[\sum_{i=1}^N (1-p_i)^N\right] \left[\sum_{i=1}^N p_i^2(1-p_i)^{N-2}\right] \tag{S5}$$

where the lhs admits an expression that suggests N_1. We can now replace those terms with relations of the size-abundances.

$$\begin{aligned} \sum_{i=1}^N (1-p_i)^N &= N_0 \\ \sum_{i=1}^N p_i(1-p_i)^{N-1} &= \frac{N_1}{\binom{N}{1}} \\ \sum_{i=1}^N p_i^2(1-p_i)^{N-2} &= \frac{N_2}{\binom{N}{2}} \end{aligned} \tag{S6}$$

which leaves

$$N_1^2 \binom{N}{2} \leq N_0 N_2 \binom{N}{1}^2. \tag{S7}$$

or

$$N_0 \geq \frac{N!}{2!(N-2)!} \frac{(N-1)!^2}{N!^2} \frac{N_1^2}{N_2}. \tag{S8}$$

which can be simplified, using $N! = N(N-1)(N-2)!$, to

$$N_0 \geq \frac{N-1}{N} \frac{N_1^2}{2N_2}. \tag{S9}$$

So that finally the Chao1 estimate of the richness including the 'unobserved' sequences can be written in terms of the number of singletons N_1, doubletons N_2, and the total sample size N as

$$\begin{aligned} R^{Chao1} &\geq R^{obs} + N_0 \\ &\geq R^{obs} + \frac{N-1}{N} \frac{N_1^2}{2N_2} \end{aligned} \tag{S10}$$

An adjustment can be made to allow $N_2 = 0$,

$$R^{Chao1*} \geq R^{obs} + \frac{N-1}{N} \frac{N_1(N_1-1)}{2(N_2+1)} \tag{S11}$$

Extended approximations for estimates of richness using higher order (tripletons etc.) frequencies have been developed [6]. A discussion of diversity inference on next generation sequencing data in the virologic context can be found in Ref. [7]. A discussion of sufficient sampling to accurately measure richness was given in Ref. [8]. We mention other estimators that are not used in the text. The jackknife 1&2 are

$$\begin{aligned} R^{j1} &= R^{obs} + \frac{N-1}{N} N_1 \\ R^{j2} &= R^{obs} + \frac{2N-3}{N} N_1 - (N-2)^2 N_2 \end{aligned} \tag{S12}$$

1.5 Chao1 confidence interval

The variance of the richness estimator can be computed following Refs. [9] and [6] as

$$var(R^{Chao1*}) = \frac{1}{4}\left(\frac{N-1}{N}\right)^2 N_1(2N_1-1)^2 + \frac{1}{2} N_1(N_1-1) - \frac{1}{4}\frac{N_1^4}{R^{Chao1*}} \tag{S13}$$

which accommodates $N_2 = 0$. An asymmetric 95% confidence interval for the richness estimate is then defined as

$$CI = \left[R^{obs} + \frac{R^{Chao1*} - R^{obs}}{c},\ R^{obs} + (R^{Chao1*} - R^{obs})c\right] \tag{S14}$$

where the factor

$$c = \exp\left[1.96\sqrt{\ln\left(1 + \frac{var(R^{Chao1*})}{(R^{Chao1*} - R^{obs})^2}\right)}\right] \tag{S15}$$

1.6 Derivation of Cauchy-Swartz inequality

The Cauchy-Swartz inequality can quickly be proved by expanding a particular double sum where i and j have the same limits,

$$\sum_i \sum_j (a_i b_j - a_j b_i)^2 = \sum_i a_i^2 \sum_j b_j^2 + \sum_i b_i^2 \sum_j a_j^2 - 2 \sum_i a_i b_i \sum_j a_j b_j. \tag{S16}$$

The lhs must be greater than zero because it is a sum of a quantity squared. Simplifying and swapping more indices

$$0 \geq 2\left[\sum_i a_i^2 \sum_i b_i^2 - \left(\sum_i a_i b_i\right)^2\right]. \tag{S17}$$

which reduces to

$$\sum_i a_i^2 \sum_i b_i^2 \geq \left(\sum_i a_i b_i\right)^2, \tag{S18}$$

i.e., Eq. S4.

1.7 Sample rarefaction

We use rarefaction curves to get a qualitative idea of how well sampled a population may be. These curves represent the average over random resamplings of the data in order to relate the sample size to the observed number of sequences. While they are only defined at integer values of sample size, we display them (as typical) as smooth curves. We interpret the result as the expected number of sequences $\langle n_k \rangle$ that would be observed given a subsample of size k. We say that we have a 'rarefied' sample'. At first, each additional subsample typically uncovers a new sequence. Thus $\langle n_k \rangle = k$. However, as sample size increases, it becomes more likely that a sequence will be repeatedly detected, and thus the curve begins to flatten $\langle n_k \rangle < k$. In our studies, we only interpolate to sample sizes below the experimental sample size, i.e. $k \leq N$. The rarefaction curve is defined

$$\langle n_k \rangle = R^{obs} - \binom{N}{k}^{-1} \sum_{r=1}^{R^{obs}} \binom{N - a(r)}{k} \tag{S19}$$

However, we do use rarefaction on our modeled distributions to assess the sample sizes that might be needed to approach the flatter part of the rarefaction curves. In that case our sample size is L, the size of the modeled population. In these calculations, $L \gg k$. Thus we can approximate the rarefaction curve expression with a substantially easier to compute variation.

We note Stirlings approximation $x! \approx \sqrt{2\pi x}(x/e)^x$ for large x. We can approximate the binomial coefficient $\binom{x}{y}$ with $x \gg y$ by using the approximation in $\frac{x!}{y!(x-y)!}$. This leaves

$$\binom{x}{y} \approx \frac{1}{y!} \left[\frac{2\pi x}{2\pi(x-y)}\right]^{1/2} \frac{x^x}{(x-y)^{x-y}} \frac{e^{x-y}}{e^x} \tag{S20}$$

cancelling terms and multiplying by $x^y x^{-y}$ allows the simplification

$$\binom{x}{y} \approx \frac{x^y e^{-y}}{y!} \left[\frac{x}{(x-y)}\right]^{1/2+x-y} \tag{S21}$$

where we can now multiply both sides by $1/x$ and use the approximation $(1+z)^{-a} \approx 1 - za$ for small z to find

$$\binom{x}{y} \approx \frac{x^y e^{-y}}{y!} \left[1 + y + y^2/x + y/2x\right] \tag{S22}$$

dropping the small terms of y/x finally results in

$$\binom{x}{y} \approx \frac{x^y}{y!} e^{-y}(1+y). \tag{S23}$$

Returning to Eq. S19 for an extrapolated distribution of total size L and richness R we have

$$\begin{aligned} \langle \tilde{n}(k) \rangle &= R - \binom{L}{k}^{-1} \sum_{r=1}^{R} \binom{L - a(r)}{k} \\ &\approx R - \left[\frac{L^k}{k!} e^{-k}(1+k)\right]^{-1} \sum_{r=1}^{R} \left[\frac{(L - a(r))^k}{k!} e^{-k}(1+k)\right] \end{aligned} \tag{S24}$$

so that the final approximation to the rarefaction curves avoids computation of large factorials,

$$\langle \tilde{n}(k) \rangle = R - \sum_{r=1}^{R} \left(1 - \frac{a(r)}{L}\right)^k. \tag{S25}$$

The extrapolated rarefaction curves based on the maximum-richness population models is discussed in the **Results** and displayed in **Supplementary Fig 4**.

1.8 Modeling rank-abundance distributions (power law)

Because the data used in our analyses (see **Fig 2**) look roughly linear on a log-log plot, we chose a power law to represent the rank abundance distributions. This decision is corroborated by similar models that accurately fit more rich uninfected T cell rank abundance distributions [10]. We include 3 variables into our model, the power law exponent α, the richness R, and the population size L. Including the population size allows us to make inferences about the distribution of unseen species. It has been noted that sample size only correlates with observed (not true) richness [11]. From that perspective, richness estimators, such as the Chao1, that employ count frequencies are the proper estimators. However, in population of known size, it should be possible to extrapolate observed data from a small sample to larger sample sizes. Indeed, true richness is bounded by true population size, for us $R \leq L$. Here, we infer an upper bound on richness based on modeling the observed rank-abundance distributions. Our model is

$$a(r; \alpha, R, L) = \left| L\psi(\alpha, R) r^{-\alpha} \right| \tag{S26}$$

where $|\cdot|$ represents rounding to the nearest integer. The normalizing constant is

$$\psi(R) = \sum_{s=1}^{R} s^{-\alpha}, \tag{S27}$$

such that

$$\sum_{r=1}^{R} a(r; \alpha, R, L) \approx L \tag{S28}$$

Note $\psi(\alpha, R)$ can be referred to as the generalized harmonic number of order R of α, or $H_{R,\alpha}$. The series converges, or the limit as R tends to infinity is finite, if $\alpha > 1$. In that limit $\psi(\alpha, R \to \infty) \to \zeta(\alpha)$, the Riemann Zeta function.

1.9 Approximating the normalizing constant by integration

We observe that certain model parameter combinations are impossible. For example, for a given power law exponent, the richness is constrained below a certain value for a given reservoir size. This observation has been considered previously in ecology under the terminology of 'feasible sets' [12]. Because the partial sum $\psi(\alpha, R)$ has no analytical solution we are aware of, we approximate the sum using an integral for large R. That is, we use

$$\psi(\alpha, R) = \sum_{s=1}^{R} s^{-\alpha} \approx \int_1^R ds\, s^{-\alpha} = \frac{R^{1-\alpha} - 1}{1-\alpha} \tag{S29}$$

to compute an approximate rank-abundance distribution

$$\tilde{a}(r) = \left| \frac{L\, r^{-\alpha}}{\psi(\alpha, R)} \right| \tag{S30}$$

Then, we solve for the maximum richness by considering the rounding process and finding the maximum first value of r for which $a(r) = 0$, which is when $L\, r_{max}^{-\alpha}/\psi(\alpha, R) \leq 1/2$. We use the condition of equality, to solve for r_{max}, which results in

$$r_{max} \approx \exp\left[-\frac{1}{\alpha} \ln\left(\frac{\psi(\alpha, R)}{2L}\right)\right]. \tag{S31}$$

Note that the expression still depends on the initial R of the model. We checked that this approximation was reasonable in our parameter ranges by directly computing the maximum richness using the rank-abundance without approximation. This comparison is shown in **Supplementary Fig 2**. Some combinations do lead to $r_{max} > L$, in which case we set $r_{max} = L$.

1.10 Model fitting procedure, example with simulated data

Here, to demonstrate the model fitting procedure, we assume we have a real distribution with underlying power-law rank abundance $\alpha = 1.5$, richness $R = 10^5$, and reservoir size $L = 10^7$. The true distribution is displayed in **Supplementary Fig 1A**. Note because of rounding, the values of richness and reservoir size are not exactly the same as our input values. The rounded-model parameters are displayed in the figure title.

To mimic an experiment we sampled 100 sequences from the true distribution using multinomial sampling with Matlab's `mnrnd`. The sampling procedure produces data for counts of each sequence based on the true rank (**Supplementary Fig 1B**). However, in the experiment, counts of zero are not observed. We 'collapse' the data by removing these zeros and re-ranking by largest to smallest count size (**Supplementary Fig 1C**). This procedure provides data that would actually be collected in the experimental setting.

In order to find the best-fit parameters of the model to recapitulate an observed rank-abundance curve we used a computational resampling procedure. In **Supplementary Fig 1D** we demonstrate 225 examples of the 2500 distributions used in the main text. We assumed a reservoir size of 10^9 cells for HIV DNA and 10^7 cells for replication competent sequences in line with previous measurements [13].

We sampled each model $n = 10$ times and formed 10 modeled cumulative proportional abundances (cpa). We scored each model with the average rss error between the model cpa (superscript m) and the experimental cpa (superscript ex) by calculating

$$\langle rss \rangle_n = \frac{1}{n} \sum_{j=1}^{n} \sqrt{\sum_{r=1}^{R} \left[cpa_j^m(r) - cpa^{ex}(r) \right]^2}. \tag{S32}$$

This procedure is illustrated against the simulated distribution in **Supplementary Fig 1E&F**. In **Supplementary Fig 1E**, the top 5 best model parameter sets are shown as compared to the simulated cpa. The model score for all possible parameter combinations is shown as a heat map in **Supplementary Fig 1F**. Because the richness appeared unidentifiable beyond a certain value, we always used the maximum richness possible for the best fit power law exponent to bias our modeling toward the more diverse reservoirs (biological ramifications are discussed in the main text).

To assess how well our model fitting performed, we illustrate a rough comparison against other possible formulations in **Supplementary Fig 1G**. Here we plot the true proportional abundance, the data sampled from that distribution and 3 fitting options. The first uses naive linear regression against log-log data. This approach systematically underestimates the true power law exponent. The second is the maximum likelihood approach detailed in Ref. [14] (and freely available for Matlab `plfit`), with $x_{min} = 1$ and finite data. The approach, denoted with the author's surnames 'CZM', systematically overestimated the power law exponent. And lastly, we illustrate our approach which we believe is a more accurate representation of the sampling procedure, and thus results in better estimates of the true power law exponent.

1.11 Other functional forms map to power-law, e.g. log-normal

Many distributions have been used previously to describe species abundance curves in the ecological literature [15, 16]. We note that the power law is inclusive of many possible choices, for instance polynomial (when $\alpha \in \mathbb{Z}$) or lognormal. Lognormal distributions simplify to power law distributions when the variance of clone size is large relative to the log-richness. To see why, we write a log-normal function for the rank-abundance distribution with log-mean $m = \langle \ln r \rangle$ and the log-standard deviation $s^2 = \langle (\ln r)^2 \rangle - \langle \ln r \rangle^2$,

$$p(r) = (\sqrt{2\pi}\sigma r)^{-1} \exp \frac{-(\ln r - m)^2}{2s^2}. \tag{S33}$$

Taking the logarithm of both sides leaves

$$\log p(r) = -\log\left(\sqrt{2\pi}s\right) + \frac{m^2}{2s^2} - \ln r \left[1 + \frac{1}{2}\left(\frac{\ln r}{s}\right)^2 + \frac{m}{s^2}\right]. \tag{S34}$$

If $s \geq \log(R)$, the order of magnitude of the variance is similar to the order of magnitude of the richness, the second term in the brackets becomes small. Defining $b = -\ln\left(\sqrt{2\pi}\sigma\right) + m^2/2s^2$ and $\alpha = 1 + m/s^2$ we then can re-exponentiate $p(r) \sim e^{b - \alpha \ln r}$ leaving

$$p(r) \sim e^b r^{-\alpha}. \tag{S35}$$

References

[1] F Maldarelli, X Wu, L Su, L Simonetti, W Shao, S Hill, J Spindler, A Ferris, J Mellors, F Kearney, J Coffin, and Hughes S. Specific HIV integration sites are linked to clonal expansion and persistence of infected cells. *Science*, 345(6193):179–183, 2014.

[2] Thor A Wagner, Sherry McLaughlin, Kavita Garg, Charles Y K Cheung, Brendan B Larsen, Sheila Styrchak, Hannah C Huang, Paul T Edlefsen, James I Mullins, and Lisa M Frenkel. Proliferation of cells with HIV integrated into cancer genes contributes to persistent infection. *Science*, 345(6196):570–573, 2014.

[3] Nina N. Hosmane, Kyungyoon J. Kwon, Katherine M. Bruner, Adam A. Capoferri, Subul Beg, Daniel I.S. Rosenbloom, Brandon F. Keele, Ya-Chi Ho, Janet D. Siliciano, and Robert F. Siliciano. Proliferation of latently infected CD4 ⁺ T cells carrying replication-competent HIV-1: Potential role in latent reservoir dynamics. *The Journal of Experimental Medicine*, 214(4):959–972, 2017.

[4] Anne Chao. Nonparametric Estimation of the Number of Classes in a Population. *Scandinavian Journal of Statistics*, 11(4):265–270, 1984.

[5] N J Gotelli and R K Colwell. Estimating species richness. In Anne E Magurran and Brian J McGill, editors, *Biological Diversity. Frontiers in Measurement and Assessment*, chapter 4, pages 39–54. Oxford University Press, Oxford UK, 2011.

[6] Chun Huo Chiu, Yi Ting Wang, Bruno A. Walther, and Anne Chao. An improved nonparametric lower bound of species richness via a modified good-turing frequency formula. *Biometrics*, 70(3):671–682, 2014.

[7] Josep Gregori, Miquel Salicrú, Esteban Domingo, Alex Sanchez, Juan I. Esteban, Francisco Rodríguez-Frías, and Josep Quer. Inference with viral quasispecies diversity indices: Clonal and NGS approaches. *Bioinformatics*, 30(8):1104–1111, 2014.

[8] Anne Chao, Robert K. Colwell, Chih Wei Lin, and Nicholas J. Gotelli. Sufficient sampling for asymptotic minimum species richness estimators. *Ecology*, 90(4):1125–1133, 2009.

[9] Anne Chao. Estimating the population size for capture-recapture data with unequal catchability. *Biometrics*, 43(4):783–91, dec 1987.

[10] Jonathan Desponds, Thierry Mora, and Aleksandra M Walczak. Fluctuating fitness shapes the clone-size distribution of immune repertoires. *Proceedings of the National Academy of Sciences of the United States of America*, 113(2):274–9, jan 2016.

[11] Amy Willis. Extrapolating abundance curves has no predictive power for estimating microbial biodiversity. *Proceedings of the National Academy of Sciences of the United States of America*, 113(35):E5096, 2016.

[12] Kenneth J. Locey and Ethan P. White. How species richness and total abundance constrain the distribution of abundance. *Ecology Letters*, 16(9):1177–1185, 2013.

[13] Ya-Chi Ho, Liang Shan, Nina N. Hosmane, Jeffrey Wang, Sarah B. Laskey, Daniel I.S. Rosenbloom, Jun Lai, Joel N. Blankson, Janet D. Siliciano, and Robert F. Siliciano. Replication-

Competent Noninduced Proviruses in the Latent Reservoir Increase Barrier to HIV-1 Cure. *Cell*, 155(3):540–551, oct 2013.

[14] Aaron Clauset, Cosma Rohilla Shalizi, and M. E. J. Newman. Power-law distributions in empirical data. *SIAM Review*, 51(4):661–703, jun 2007.

[15] Anne Chao. Species Estimation and Applications. *Encyclopedia of Statistical Sciences*, 1(1):7907–7916, 2004.

[16] Brian J. McGill, Rampal S. Etienne, John S. Gray, David Alonso, Marti J. Anderson, Habtamu Kassa Benecha, Maria Dornelas, Brian J. Enquist, Jessica L. Green, Fangliang He, Allen H. Hurlbert, Anne E. Magurran, Pablo A. Marquet, Brian A. Maurer, Annette Ostling, Candan U. Soykan, Karl I. Ugland, and Ethan P. White. Species abundance distributions: Moving beyond single prediction theories to integration within an ecological framework. *Ecology Letters*, 10(10):995–1015, 2007.

2 Supplementary Figures

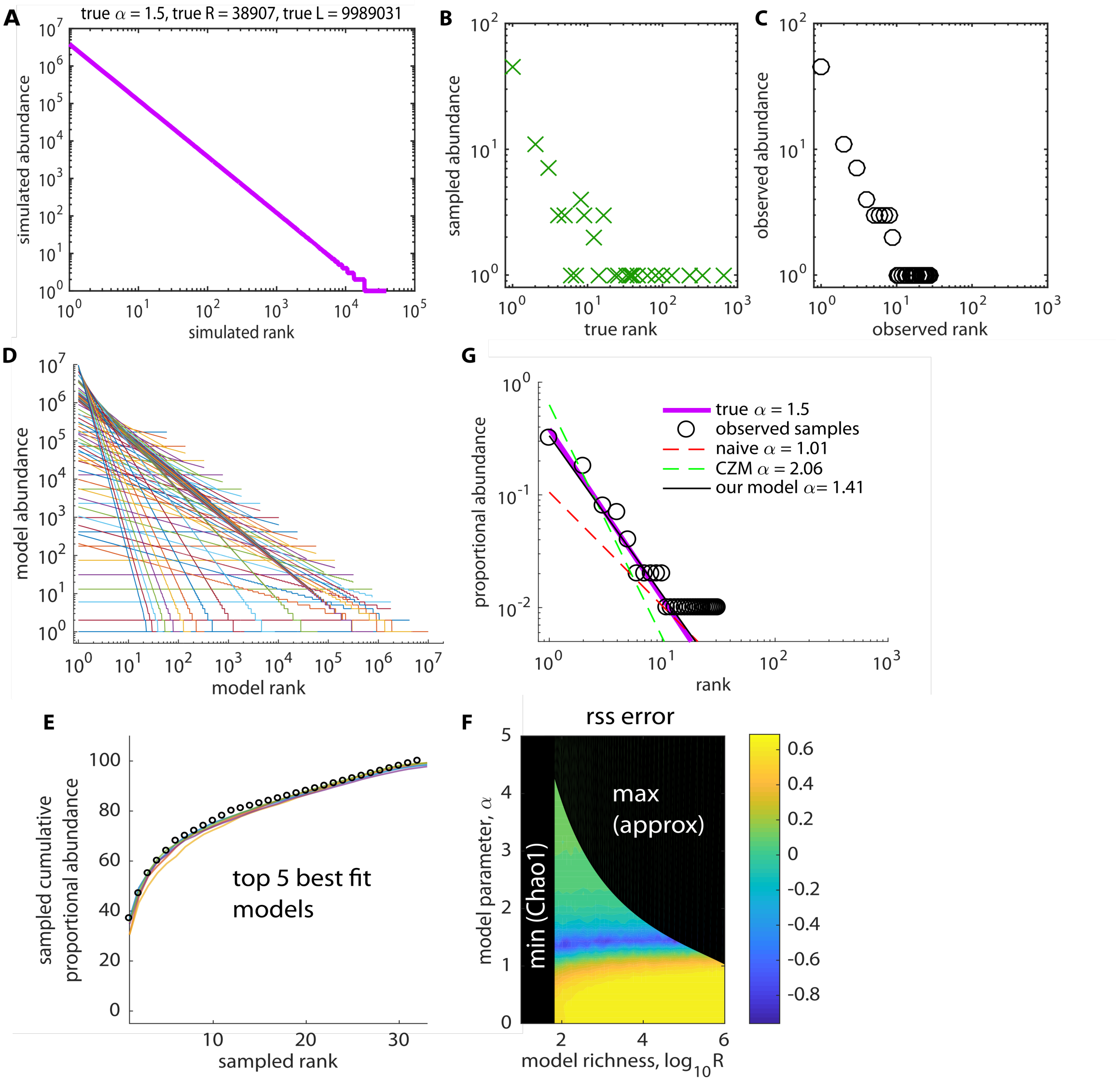

Supplementary Fig 1. A) Simulating a true distribution assuming underlying power-law $\alpha = 1.5$. B) Sampling from the true distribution to generate simulated data. C) By re-ranking the data and removing all zero counts, we develop a surrogate data set. D) 225 examples (out of 2500 total) distributions used in the fitting procedure ($\alpha = 0 \rightarrow 5$ and $R = 10^{1\rightarrow 7}$). E) The top 5 model cpas (colored lines) and simulated data cpa (black circles). F) A heat map of the avg rss error from 10 replicate samplings demonstrates the model score for each of the parameter combinations. The large black areas represent constraints on richness derived from the Chao1 estimator (lower bound) and the approximate integral $\psi(R)$ (upper bound). Note that the minimum error specifies a power-law exponent close to the true value of $\alpha = 1.5$ but does not identify a unique value for the richness. In our extrapolations in the text, we use the maximum allowable richness to bias the model toward higher diversity and less proliferation. G) Comparison of true power law, observed sampled data and three fitting methods. Naive log-log-linear regression and CZM maximum likelihood (authors in [14]) under- and over-estimate the true value, respectively. Our modeling approach provides the most accurate power-law exponent given this type of data.

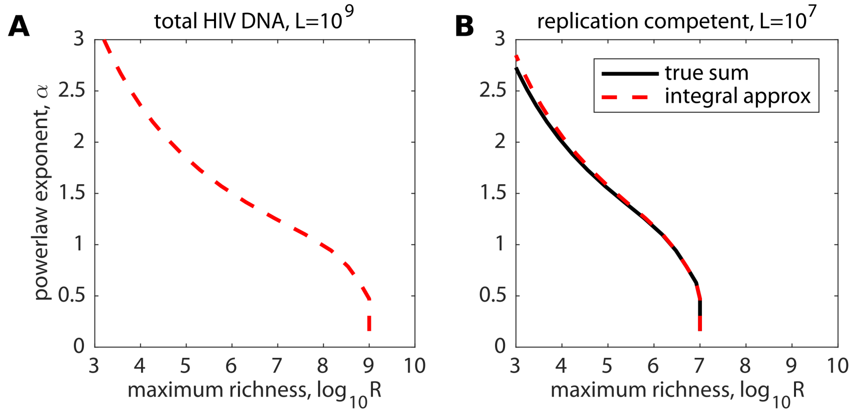

Supplementary Fig 2. The maximum richness is constrained by power law exponent α and total reservoir size L. A) total HIV DNA approximation. B) replication competent HIV DNA approximation compared with numerically calculating r for which $0 = |L \sum_{s=1}^{r} s^{-\alpha}|$.

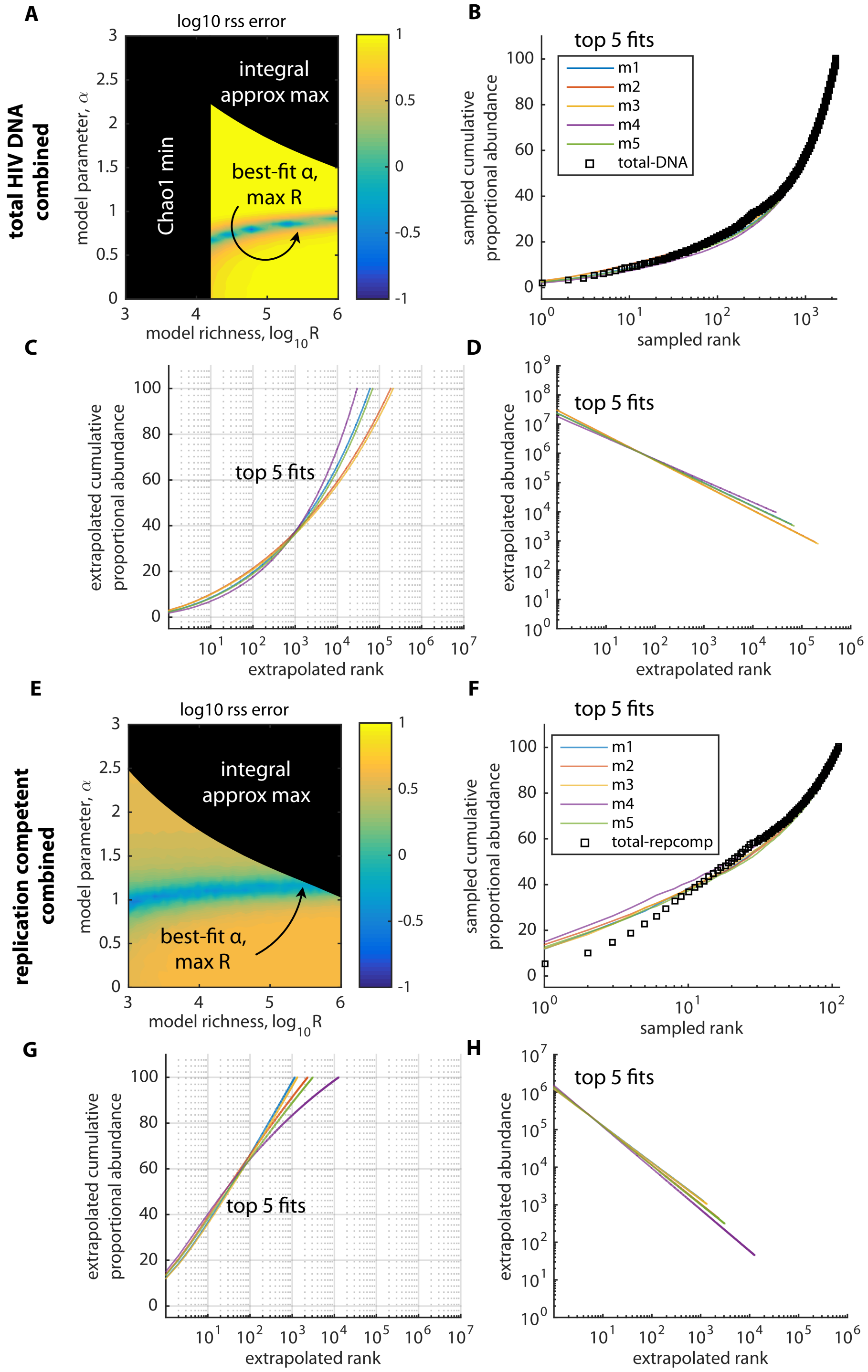

Supplementary Fig 3. Fitting results and extrapolated model distributions using compiled sequence abundance from **Fig 2** in the main text. A-D) total HIV DNA integration site data and E-H) replication competent DNA sequences. From left to right, panels indicate A&E) the fitting results for a range of α and R, B&F) the data (black circles) and the top 5 best fit models, C&G) the extrapolated cumulative proportional abundance, and D&H) the extrapolated rank abundance.

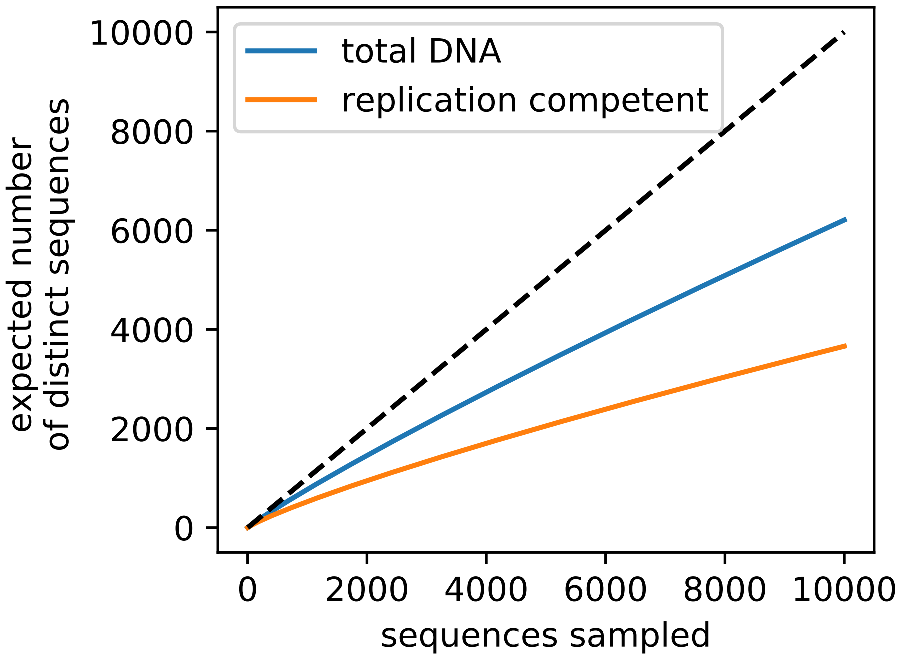

Supplementary Fig 4. The extrapolated rarefaction curves using the maximum richness estimated power law models from the combined data in **Supplementary Fig 3** above.
